# Involvement of interleukin-1β in the autophagic process of microglia: relevance to Alzheimer’s disease

**DOI:** 10.1186/1742-2094-10-151

**Published:** 2013-12-13

**Authors:** Arnaud François, Faraj Terro, Thierry Janet, Agnès Rioux Bilan, Marc Paccalin, Guylène Page

**Affiliations:** 1 EA3808 Molecular Targets and Therapeutic of Alzheimer’s Disease, University of Poitiers, Poitiers F-86022, France; 2 Faculty of Medicine, Laboratory of Histology and Molecular Biology, University of Limoges, Limoges F-87025 France; 3 Service d’Histologie et de Cytogénétique, Hôpital de la Mère et de l’Enfant, Limoges F-87025, France; 4 Service de Gériatrie, Poitiers University Hospital, Poitiers F-86021, France; 5 CMRR, Poitiers University Hospital, Poitiers F-86021, France; 6 CIC-P 0802, Poitiers University Hospital, Poitiers F-86021, France; 7 UFR Médecine et Pharmacie, 6 rue de la Milétrie, BP 199, 86034 Poitiers, Cedex, France

**Keywords:** Inflammation, Autophagy, Alzheimer, Microglia, IL-1β

## Abstract

**Background:**

Autophagy is a major pathway of protein and organelle degradation in the lysosome. Autophagy exists at basal constitutive level and can be induced as a defense mechanism under stress conditions. Molecular relationships between autophagy and inflammation at the periphery were recently evidenced, highlighting a role of autophagy in the regulation of inflammation. Impairment of autophagy (with accumulation of autophagic vacuoles) and substantial inflammation are found in neurodegenerative diseases such as Alzheimer’s Disease (AD). However, the links between autophagy and inflammation in AD remain to be determined.

**Methods:**

Here, we examined the inflammatory reaction and autophagy in murine tri-cultures of neurons, astrocytes, and microglia. Tri-cultures were exposed to various inflammatory stresses (lipopolysaccharide (LPS), amyloid peptide (Aβ42) with or without cytokines) for 48 hours. Furthermore, the relationships between inflammation and autophagy were also analyzed in astrocyte- and microglia-enriched cultures. Data for multiple variable comparisons were analyzed by a one-way ANOVA followed by a Newman-keuls’ test.

**Results:**

Aβ42 induced a low inflammation without accumulation of acidic vesicles contrary to moderate or severe inflammation induced by LPS or the cytokine cocktail (IL-1β, TNF-α, and IL-6) or IL-1β alone which led to co-localization of p62 and LC3, two markers of autophagy, with acidic vesicles stained with Lyso-ID Red dye. Moreover, the study reveals a major role of IL-1β in the induction of autophagy in tri-cultures in the presence or absence of Aβ42. However, the vulnerability of the autophagic process in purified microglia to IL-1β was prevented by Aβ42.

**Conclusion:**

These findings show a close relationship between inflammation and autophagy, in particular a major role of IL-1β in the induction of the microglial autophagy which could be the case in AD. New therapeutic strategies could target inflammasome and autophagy in microglia to maintain its role in the amyloid immunosurveillance.

## Background

Macroautophagy (hereafter ‘autophagy’), a basal housekeeping process, delivers a wide spectrum of cytosolic substrates including long-lived proteins, protein aggregates, and organelles to lysosomes for subsequent degradation. In addition to its role in cellular homeostasis, autophagy plays an essential role in the development of innate and adaptive immune responses and in physiological responses to exercise and aging. Autophagy can also be induced by various environmental and cellular stresses, such as nutrient starvation, trophic factor withdrawal, and immune stimuli [1]. Autophagy is mainly regulated by two key kinases and nutrient sensors: the mammalian target of rapamycin (mTOR), a Ser/Thr kinase which inhibits autophagy, and AMP-activated protein kinase (AMPK), a positive regulator of autophagy [2].

Dysfunctions of autophagy are widely implicated in pathological conditions, including cancer, metabolic and neurodegenerative disorders, and cardiovascular and pulmonary diseases [3]. However, molecular mechanisms underlying these connections are not completely elucidated. Downregulation of both IFN responses to viral infection and proinflammatory cytokine responses to invading pathogens and the inhibition of inflammasome-dependent maturation and secretion of proinflammatory cytokines (for example, IL-1β) have been demonstrated [4-6]. The first link between autophagy and inflammation was established by the discovery that the inflammatory Crohn’s disease is linked to the mutations of Atg16L1, a key component of the core machinery of autophagy. Following stimulation by lipopolysaccharide (LPS), autophagy-related protein 16-1 (Atg16L1)-deficient macrophages produce high amounts of the proinflammatory cytokines IL-1β and IL-18, suggesting that autophagy might have an inhibitory effect on the maturation and secretion of proinflammatory cytokines [7].

Currently, no studies have examined the potential links between autophagy and inflammation activation in the context of central nervous system (CNS) disease. At early stages of Alzheimer’s disease (AD), autophagy is induced in vulnerable neurons of AD patients and in a transgenic mouse model [8,9]. A reduced expression of the autophagy-related protein Beclin-1, which is required for the early step of autophagosome formation [10], and co-localization of sequestosome 1/p62 (an adaptor protein with several functional domains to target polyubiquitinated protein cargo to autophagosomes), ubiquitin, and hyperphosphorylated tau in aggregates have been reported in the cortex and hippocampus of AD patients [11,12]. Furthermore, in familial AD, autophagosomes proliferated and the level of LC3-II (a marker of autophagosome) increased [13]. In addition, components required for the generation of Aβ (amyloid precursor protein (APP), presenilin 1 (PS1), nicastrin, and β-secretase) are found in autophagic vacuoles (AVs) [14].

Besides, several lines of evidence suggest that inflammation plays a key role in the pathogenesis of AD [15]. Recently, amyloid-β-induced activation of the NLRP3 inflammasome was demonstrated to enhance AD progression by mediating harmful chronic inflammation tissue response [16].

In the present study, we set out to investigate the interactions between autophagy and inflammation in AD. Firstly, in primary tri-cultures of neurons, astrocytes, and microglia, we showed that an inflammatory stress, particularly driven by IL-1β, induced autophagy with acidic vesicle accumulation contrary to amyloid peptide (Aβ42). Immunolabeling in tri-cultures showed that only microglia displayed an accumulation of acidic vesicles as demonstrated in Lyso-ID Red-stained cells under inflammatory stress. Secondly, on purified cultures of astrocytes or microglia, we confirmed that only in microglia the autophagic process was sensitive to IL-1β, while Aβ42 prevented the accumulation of acidic vesicles. These close relationships between inflammasome and autophagy in the AD model could open new therapeutic strategies targeting microglia to maintain its role in the amyloid immunosurveillance.

## Methods

### Chemical products

Sodium fluoride (NaF), phenylmethylsulfonyl fluoride (PMSF), protease and phosphatase inhibitor cocktails, dithiothreitol (DTT), 0.01% poly-L-lysine (PLL) solution, Percoll, sterile filtered dimethyl sulfoxide (DMSO) Hybri-Max, Triton X-100, paraformaldehyde (PFA), and all reagent-grade chemicals for buffers were purchased from Sigma-Aldrich (Saint-Quentin Fallavier, France). Dulbecco’s modified Eagle’s medium (DMEM) (1 g/L and 4.5 g/L glucose), F-12, minimum essential medium Eagle (MEM) and neurobasal media, B-27 supplement, 200 mM L-glutamine, 5,000 units of penicillin (base) and 5,000 μg of streptomycin (base)/mL (penicillin-streptomycin (PS)) mixture, 0.05% trypsin-EDTA (1X) phenol red, FBS certified, horse serum, NuPAGE Novex 3-8% Tris-Acetate and Novex 4-20% Tris-Glycine gels, NuPAGE LDS Sample Buffer (4X), NuPAGE Sample Reducing Agent (10X), Novex Tris-Glycine and NuPAGE Tris-Acetate SDS Running Buffer, NuPAGE Antioxidant, iBlot transfer stack regular (nitrocellulose), and the ProLong Gold antifade reagent with 4′,6-diamidino-2-phenylindole (DAPI) were purchased from Gibco-Invitrogen (Fisher Scientific distributor, Illkirch, France). The β-amyloid peptide (Aβ42) and imidazole-oxindole compound C16 were purchased from Merck Chemicals Calbiochem (Merck Millipore, Billerica, MA, USA). Primary antibodies and secondary anti-rabbit IgG antibody conjugated with horseradish peroxidase (HRP) and recombinant cytokines (TNF-α, IL-1β, and IL-6) were purchased from Cell Signalling (Ozyme, Saint-Quentin-en-Yvelines, France) except LC3 and p62/SQMT1 from MBL (CliniSciences distributor, Nanterre, France), anti-β actin from Sigma-Aldrich, and HRP conjugated anti-mouse IgG from Fisher Scientific. For immunofluorescence, mouse anti-glial fibrillary acidic protein (GFAP) antibodies were purchased from Cell Signalling, chicken anti-microtubule-associated protein 2 (MAP2) from Abcam (Paris, France), rat anti-macrosialin (murine homologue of the human CD68) from AbD Serotec (Düsseldorf, Germany), and secondary antibodies: swine anti-rabbit fluorescein isothiocyanate (FITC) and tetramethylrhodamine isothiocyanate (TRITC) from DakoCytomation (Trappes, France), goat anti-mouse Alexa 647 from Invitrogen (Fisher Bioblock Scientific distributor, Illkirch, France), goat anti-chicken-FITC from Abcam (Paris, France), and IgG- and protease-free bovine serum albumin (BSA) from Jackson ImmunoResearch Europe, Ltd (Beckman-Coulter distributor, Villepinte, France).

### Murine primary cultures

The experiments using animals have made a request to the local ethics committee according to “La direction départementale de la protection de la population (DDPP)” protocol was registered under the number 07.12. Any experimental research on animals followed internationally recognized guidelines.

#### Neurons/astrocytes/microglia

The tri-culture was obtained as previously described [17]. First, primary glial cultures were prepared from B6C3F1 mouse embryos of 18 days. Briefly, brains were quickly removed, and cerebral cortico-hippocampal regions were dissected. Cells were then mechanically dissociated and suspended into DMEM (1 g/L glucose)/F12 (1:1) with 10% FBS/1% of antibiotic PS, seeded at a density of 4 × 10^5^ cells/mL in Nunc EasYFlask (75 cm^2^) coated with 0.001% PLL, and then incubated at 37°C in a humidified 5% CO_2_ atmosphere. After 14-day-old cultures, microglia was purified.

Second, primary cultures with neurons and astrocytes were prepared from the cortex and hippocampus of B6C3F1 mouse embryos of 18 days as indicated above. Cells were suspended in MEM/neurobasal (1:1) supplied with 18 mM glucose, 2% B-27 supplement, 1% glutamine, 2.5% FBS, 2.5% horse serum, and 1% PS, and seeded in 6-well plates (8.25 × 10^5^ cells per well) coated with 0.001% PLL. Cultures were then maintained at 37°C in a humidified 5% CO_2_ atmosphere. At day 5, neurons and astrocytes were cultured with microglia purified from the primary glial culture described above.

Third, microglia was purified from glial cultures on day 14 on the Percoll gradient as previously described [17]. Purified microglia was added at the proportion of 15% of the initial density of 5-day-old primary neuron and astrocyte cultures. The obtained tri-cultures were then used 3 days later in the experimental conditions.

#### Astrocyte-enriched cultures

Primary astrocyte cultures were prepared from the cortex and hippocampus of B6C3F1 mice aged 2 days. Cells were suspended in DMEM (4.5 g/L glucose), 1% glutamine, 10% FBS, 1% PS, and seeded in 6-well plates (8.25 × 10^5^ cells per well) coated with 0.001% PLL. Cultures were then maintained at 37°C in a humidified 5% CO_2_ atmosphere. These cells were used 8 days later in experiments.

#### Microglial cell-enriched cultures

Primary microglia cultures were prepared as described above and purified microglia were seeded in 6-well plates (3.75 × 10^5^ cells per well) coated with 0.001% PLL. Cultures were then maintained at 37°C in a humidified 5% CO_2_ atmosphere and used 3 days later in experiments. The enrichment of microglial cells in these cultures was approximately 79 ± 10% of microglia (n = 5 in duplicate, five fields per coverslip).

### Culture treatments

Tri-cultures, purified astrocytes, and microglia cultures were treated with either C16 (a specific inhibitor of double-stranded RNA-dependent protein kinase (PKR)) at 210 nM or 0.2% DMSO (vehicle of C16), in serum-free MEM:neurobasal (1:1)/1% glutamine/1% PS medium for tri-cultures, serum-free DMEM, 1% glutamine, 1% PS for astrocytes, and serum-free DMEM/F12 (1:1)/1% PS for microglia, 1 hour before inflammatory stress. The concentration of C16 was chosen as previously described in these primary tri-cultures [17]. All cultures were exposed to different inflammatory stresses for 48 hours: Aβ42 used at 20 μM and was previously incubated for 72 hours at 37°C for aggregation as recommended by the Merck Chemical supplier (this Aβ42 solution contains monomers at 4 kDa, oligomers at 8 and 12 kDa, and fibrils as described by Couturier *et al*. [17]); LPS used at 100 ng/mL; and mouse recombinant TNF-α, IL-1β, or IL-6 cytokines used at 200 pg/mL alone or in combination (‘cocktail’ condition). After 24 hours, cultures were treated with an autophagic inhibitor, bafilomycin A1 (Baf), at 50 nM for 24 hours.

### Cell lysis

After 48 hours of treatment, media were stored at -80°C until used for xMAP (Luminex, Austin, TX, USA) assays. Cells were then washed with PBS (1.54 mM KH_2_PO_4_, 154 mM NaCl, and 2.71 mM Na_2_HPO_4_-7H_2_O) and lysed in ice-cold lysis buffer (50 mM Tris-HCl, 50 mM NaCl, pH 6.8, 1% (v/v) Triton X-100, 1 mM PMSF, 50 mM NaF, 1% (v/v) protease inhibitor, and 1% (v/v) phosphatase inhibitor cocktail). Lysates were sonicated for 10 seconds and centrifuged at 15,000 × *g* for 15 minutes at 4°C. The supernatants were collected and analyzed for protein determination using a Qubit protein assay kit (Fisher Scientific). Samples were frozen at -80°C until further analysis.

### Immunoblotting

Samples (40 μg protein of cell lysates) were prepared for electrophoresis by adding NuPAGE LDS Sample Buffer (4X) and NuPAGE Sample Reducing Agent (10X). After heating, samples were loaded into NuPAGE Novex 4-20% Tris-Glycine Mini Gels, run at 150 V for 60 minutes in Novex Tri- Glycine SDS Running Buffer containing NuPAGE Antioxidant and in NuPAGE 3-8% Tris-Acetate Gels, run at 125 V for 120 minutes in Novex Tris-Acetate SDS Running Buffer containing NuPAGE Antioxidant. Gels were transferred to nitrocellulose membranes using the iBlot Dry Blotting System set to program 20 V for 7 minutes. Membranes were washed for 10 minutes in Tris-buffered saline and Tween (TBST) (20 mM Tris-HCl, 150 mM NaCl, pH 7.5, and 0.05% Tween 20) and blocked for 2 hours in TBST containing 5% BSA. Blots were incubated with primary antibody in blocking buffer overnight at 4°C. Antibodies used were rabbit anti-Beclin-1 (1:500), rabbit anti-p62/SQMST1 (1:500), rabbit anti-LC3 (1:500), rabbit anti-mTOR (1:500), rabbit anti-P_S2448_ mTOR (1:500), rabbit anti-p70S6K (1:500), and rabbit anti-P_T389_-p70S6K (1:500). Membranes were washed twice with TBST and then incubated with the peroxidase conjugated secondary anti-rabbit antibody (1:1,000) for 1 hour at room temperature. Membranes were washed again and exposed to the chemiluminescence Luminata Forte Western HRP Substrate (Millipore, Saint-Quentin-en-Yvelines, France) followed by signal capture with the Gbox system (GeneSnap software, Syngene, Ozyme distributor). After two washes in TBST, membranes were probed with mouse antibody against β-actin (1:100,000) overnight at 4°C. They were then washed with TBST, incubated with peroxidase conjugated secondary anti-mouse antibody (1:1,000) for 1 hour, exposed to the chemiluminescence Luminata Classico Western HRP Substrate, and signals were captured. Automatic image analysis software was supplied with GeneTools (Syngene). Ratios of protein/β-actin and phosphorylated protein/total protein (represented activation of kinase) were calculated and shown in the corresponding figures.

### Luminex xMAP assay

Mouse cytokine Luminex 3-plex kits (for TNF-α, IL-1β, and IL6) were purchased from Millipore. The assay was performed in 96-well plates and all reagents and plates were prepared according to the manufacturers’ instructions. Each standard (25 μL) from a range of concentrations (10,000 to 3.2 pg/mL) (assay buffer was used as a blank), quality controls, and samples were added to the relevant wells. The culture media and cell lysis buffer were added as background controls. The mixed bead solution was sonicated and vortexed prior to adding 25 μL into each well. The plates were sealed and incubated with agitation on a plate shaker at 750 rpm (Titrimax, Fisher Scientific) overnight at 4°C in a darkroom. Plates were washed twice with 200 μL assay wash buffer, and 25 μL biotinylated detection antibodies were added per well. The samples were incubated for 1 hour at room temperature on the plate shaker at 750 rpm in a darkroom. Without washing, 25 μL/well of streptavidin-phycoerythrin solution was added, and plates were incubated for another 30 minutes at room temperature on a plate shaker at 750 rpm in a darkroom. After staining was complete, the microbeads were washed twice with 200 μL/well wash buffer. The microbeads were resuspended in 150 μL/well of Luminex Sheath Fluid on a plate shaker at 500 rpm for 5 minutes at room temperature before analyzing. The assay was acquired on a Luminex 200 instrument using xPONENT software (Luminex). An acquisition gate of between 8,000 and 15,000 was set to discriminate against any doublet events and ensure that only single microbeads were measured. A total of 50 beads/well were collected and median fluorescence intensities (MFIs) were measured. The sensitivity limit was 5.4, 1.1, and 2.3 pg/mL for IL-1β, IL-6, and TNFα, respectively. The MFIs were converted to concentrations using the best parameter logistic fit curve generated for each analyte from the six standards using Milliplex Analyst software (Millipore). Results were expressed as pg/mL for culture media and pg/mg protein for cell lysates.

### Confocal immunocytofluorescence

After treatment, cells were washed with PBS and fixed with 4% PFA for 15 minutes at room temperature. After three washes with PBS, the permeabilizing and blocking PBS buffer (137 mM NaCl, 2.7 mM KCl, 1.7 mM KH_2_PO_4_, 10.14 mM Na_2_HPO_4_, pH 7.4, containing 0.3% triton X-100 and 5% BSA) was added for 1 hour at room temperature.

In tri-cultures, staining of neurons, astrocytes, microglia, and autophagosomes was performed by incubating coverslips overnight at 4°C with a mix containing chicken anti-MAP2 (1:50), mouse anti-GFAP (1:100) with a rabbit anti-p62 (1:50) or rabbit anti-LC3 (1:50), or a mix with rat anti-CD68-R-phycoerythrin (RPE) (1:25), mouse anti-GFAP (1:100) with a rabbit anti-p62 or rabbit anti-LC3 (1:25) in PBS containing 0.3% triton X-100 and 1% BSA. In purified primary microglia, a mix solution containing rat anti-CD68-RPE (1:25), mouse anti-GFAP (1:100) with a rabbit anti-p62 (1:50) or rabbit anti-LC3 (1:50) was used. Cells were then rinsed twice with PBS before 1 hour of incubation at room temperature either with a mix containing swine anti-rabbit TRITC (1:20) for p62 or LC3, goat anti-chicken FITC (1:50) for MAP2, goat anti-mouse Alexa Fluor 647 (1:25) for GFAP to study p62 or LC3 expression in neurons and astrocytes of tri-cultures, or a mix containing swine anti-rabbit FITC (1:30) for p62 or LC3, goat anti-mouse Alexa Fluor 647 (1:25) for GFAP to study p62 or LC3 expression in astrocytes and microglia of tri-cultures and in purified microglia. Finally, cells were washed twice in PBS and twice in distilled water before mounting with the ProLong Gold antifade reagent with DAPI.

### Lysosome activity assessment

In order to detect lysosome and lysosome-like organelle perturbations in our experimental conditions, we used Lyso-ID Red Cytotoxicity Kit (GFP-Certified) (Enzo Life Sciences, Exeter, UK) for 96-well microplates. According to this assay, an increase in the red lysosome signal indicates the accumulation of Lyso-ID Red dye within the cells reflecting an increase in lysosome or lysosome-like vesicle size and/or number. However, quantification of fluorescence was not performed because in our experimental conditions all cells were not fluorescent and thus the fluorescent intensity was under the limit of detection contrary to the positive control with verapamil, a cationic amphiphilic drug, known to cause phospholipidosis, the accumulation of phospholipids in acidic organelles in the cells, and used by the manufacturer to validate the assay. Thus, after treatment, tri-cultures or purified microglia were washed once with 100 μL of 1X assay buffer and incubated with 100 μL of the 1X Lyso-ID Red reagent in a darkroom for 30 minutes at room temperature. Then, cells were washed twice with 200 μL of 1X assay buffer per wash and fixed with 4% PFA for immunocytofluorescence as described above to study co-staining with p62 and LC3.

Multiple labeled samples were examined with a spectral confocal FV1000 station installed on an inverted microscope IX-81 (Olympus, Tokyo, Japan) with Olympus X40 oil, 1.2 NA, and an objective lens with optical section separation (z-interval) of 0.6 μm. Fluorescence signal collection, image construction, and scaling were performed using the control software (Fluoview FV-AS10, Olympus). Multiple fluorescence signals were acquired sequentially to avoid cross-talk between image channels. Fluorophores were excited with a 405 nm line of a diode (for DAPI), 488 nm line of an argon laser (for Alexa Fluor 488 or FITC), 543 nm line of a helium–neon laser (for TRITC and RPE), and 633 nm line of a helium–neon laser (for Alexa Fluor 647). Emitted fluorescence was detected through spectral detection channels between 425 to 475 nm and 500 to 530 nm, for blue and green fluorescence, respectively, and through 560 nm and 650 nm long pass filters for red and far red fluorescence, respectively. The images then were merged as an RGB image. Signals were analyzed by ImageJ software (National Institutes of Health, Bethesda, MD, USA). For each channel in the merged image, the intensity of signal in neurons, astrocytes, and microglia were analyzed by enclosing cells using the tools available in the software.

Co-labeling analyses between Lyso-ID and p62 or LC3 were performed by using Imaris software (version 7.4.2, Bitplane, Zurich, Switzerland) in a region of interest (ROI). Two-dimensional fluorograms displayed the intensity and distribution of different colored pixels within the merged image as a scattergram generated for each analysis.

### Statistical analysis

Results are expressed as means ± SEM. Data for multiple variable comparisons were analyzed by a one-way ANOVA followed by a Newman-Keuls’ test as a *post hoc* test using the statistical program GraphPad InStat (GraphPad Software, San Diego, CA, USA). The level of significance was *P* <0.05.

## Results

At first, we studied the impact of inflammatory stress on autophagy in tri-cultures of neurons, astrocytes, and microglia, a model previously published [17]. This model brings together the major cellular players of the CNS including neurons (36%), astrocytes (57%), and microglia (6%), and seems necessary to answer our question, taking into account the cellular and molecular responses of these three cell types in the same environment that models the CNS.

### Levels of cytokines in primary tri-cultures exposed to LPS or Aβ42

LPS is the major component of the outer membrane of gram-negative bacteria and is well reported to induce inflammatory response and cytokine production through the NF-κB signaling pathway [18,19]. C16 is a specific imidazolo-oxindole inhibitor of PKR and can prevent the NF-κB signaling pathway and cytokine production [17,20-22].

**Figure 1:**
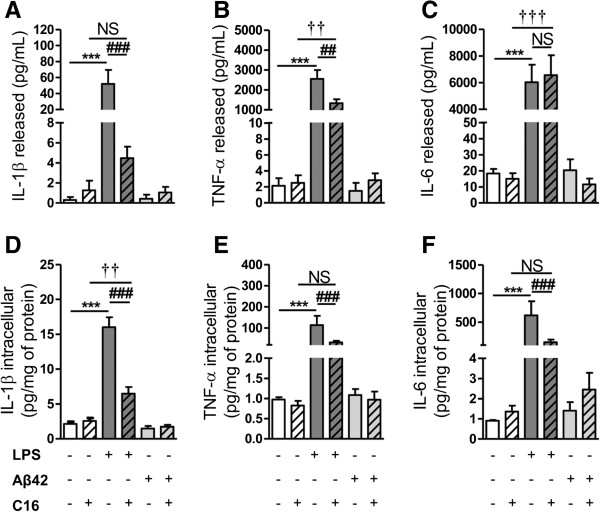
**Cytokine levels induced by LPS or Aβ42 in primary tri-cultures.** Cytokine (IL1-β, TNF-α, and IL-6) levels in culture medium (**A**, **B**, **C**, respectively) and in cell lysates (**D**, **E**, **F**, respectively) of tri-cultures pre-treated or not with 210 nM C16 and treated with 100 ng/mL LPS or with 20 μM Aβ42 in serum-free medium were analyzed by the 3-plex Luminex xMAP assay containing a mixture of specific beads for each cytokine as described in the Methods section. Cytokine levels in culture medium and cell lysates are expressed in pg/mL and pg/mg protein, respectively. Results are mean ± SEM for six experiments in duplicate. ^***^P <0.001 compared to control; ^##^*P* <0.01, ^###^*P* <0.001 compared to LPS; ^††^*P* <0.01, ^†††^*P* <0.001 compared to C16 by one-way ANOVA with a Newman-Keuls multiple comparison test. LPS, lipopolysaccharide; NS, not significant.

As expected, 48 hours of treatment of tri-cultures of neurons/astrocytes/microglia with 100 ng/mL LPS induced a substantial increase in the levels of intracellular and released cytokine (Figure 1). Intracellular and released IL-1β levels were 7.5 and 177 times higher, respectively, compared to control values (Figure 1A,D). TNF-α levels were increased by a factor of 113 and 1,200, respectively (Figure 1B,E) and IL-6 levels were increased by a factor of 619 and 330, respectively (Figure 1C,F).

Pre-treatment with 210 nM C16 significantly decreased levels of LPS-induced cytokines except for released IL-6. Intracellular levels of all cytokines were decreased by 70% to 80% (Figure 1D,E,F) and reductions in TNF-α and IL-1β in the extracellular medium were 50% and 90%, respectively (Figure 1A,B). Interestingly, the prevention of LPS-induced cytokine levels was complete for released IL-1β (Figure 1A) and TNF-α and IL-6 in cell lysates (Figure 1E,F, respectively), but partial for IL-1β in cell lysates and released TNF-α (Figure 1B,D). C16 failed to prevent LPS-induced increase of released IL-6 (Figure 1C).

Exposure of tri-cultures to Aβ42 20 μM for 48 hours induced a very low inflammation stress of less than 10 μg/mg protein and 20 pg/mL in cell lysates and extracellular medium, respectively. These values are comparable to those observed in controls (in the presence or absence of C16) (Figure 1).

### Monitoring of the autophagic flux in primary tri-cultures exposed to LPS or Aβ42

To determine whether autophagy changes occur during an inflammatory stress, Beclin-1, p62, LC3-I, and LC3-II were investigated. Beclin-1 is a key component of the class III phosphatidylinositide 3-kinase (PI3K) complex which is involved in the initiation of autophagosome formation [23]; p62 is an autophagic receptor which recognizes ubiquitinylated proteins and interacts with LC3-II at the forming autophagosome [24]; LC3 is present in a free cytoplasmic form as LC3-I which when it is associated with phosphatidylethanolamine (through an ubiquitin-like conjugation reaction) of the membrane of autophagosome produces LC3-II, a useful marker of autophagic membranes. LC3-II migrates to an apparently lower Mr position (LC3-II) by electrophoresis [24]. Both p62 and LC3-II are degraded with ubiquitinylated protein after autophagosome fusion with lysosome. To understand whether autophagy was impaired in our experimental conditions, an autophagic flux inhibitor, Baf, has been used in particular to detect LC3-II which is difficult to quantify during autophagic flux. This toxin blocks the lysosome acidification required for the fusion with autophagic vacuole by specific inhibition of the vacuolar-type H^+^-ATPase lysosomal pump [25]. It should be noted that Baf did not modify LPS-induced increases in cytokines. Furthermore, in the presence of Baf, C16 partially reduced levels of all intracellular cytokines (46% to 56% of inhibition) and of extracellular TNF-α and IL-1β (37% and 73%, respectively) except for released IL-6 [see Additional file 1].

**Figure 2:**
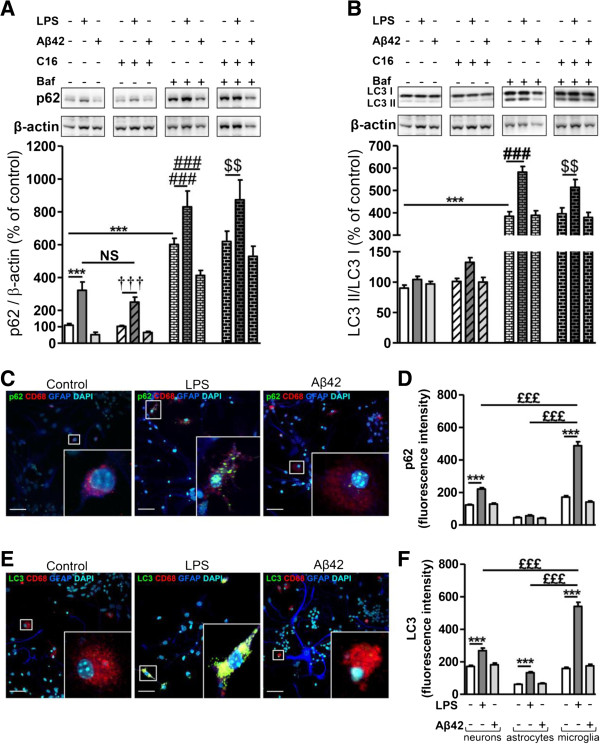
**Changes in autophagic factors after exposure to LPS or Aβ42 in primary tri-cultures.** Representative immunoblots show the immunoreactivity of **(A)** p62, **(B)** LC3-I, LC3-II, and β-actin from cell lysates of primary tri-cultures exposed to 20 μM Aβ42 or 100 ng/mL LPS pretreated or not with 210 nM C16 in serum-free medium for 48 hours, treated or not with an autophagic flux inhibitor bafilomycin A1 (Baf) at 50 nM for 24 hours before cell lysis. Densities were quantified using GeneTools software. Data of each protein were reported to data of the corresponding β-actin. The results are expressed as arbitrary units (percentage of the control set at 100%) and are mean ± SEM from six independent experiments in duplicate. Co-labeling of autophagic receptor **(C)** p62 (green) or **(E)** LC3 (green), CD68 for microglia (red), GFAP for astrocytes (blue), and DAPI for nuclei (cyan) in tri-cultures seeded on coverslips. All images were from a compilation of the entire z-series sections acquired by confocal microscopy (Olympus IX-81). A white square represents a magnified ROI. Scale bars, 42 μm. Fluorescence intensity of **(D)** p62 and **(F)** LC3 staining in neurons, astrocytes, and microglia quantified using ImageJ software in confocal images. ^***^P <0.001 compared to control; ^†††^*P* <0.001 compared to C16; ^##^*P* <0.01, ^###^*P* <0.001 compared to Baf; ^$$^*P* <0.01 compared to Baf with C16; ^£££^*P* <0.001 compared to LPS treatment in microglia by one-way ANOVA with a Newman-Keuls multiple comparison test. Baf, bafilomycin A1; DAPI, 4′,6-diamidino-2-phenylindole; GFAP, glial fibrillary acidic protein; LPS, lipopolysaccharide; NS, not significant; ROI, region of interest.

As expected, LPS-treated tri-cultures displayed a very reactive microglia, marked by a larger cell body and numerous radiating cytoplasmic projections (Figure 2C,E). LPS clearly affected neuron viability which is manifested by the presence of highly condensed nuclei and the absence/retraction of neurites. Astrocytes were protoplasmic but some were stellar [see Additional file 2]. Conversely, in control or Aβ42 conditions, neurons had long processes in communication with others, microglia remained ‘resting’, and astrocytes drew a very protoplasmic layer of cells (Figure 2C,E) [see Additional file 2].

The expression of p62 was significantly increased by LPS treatment (194% increase) but C16 failed to reverse this increase (Figure 2A). Blockade of the autophagic flux by Baf increased p62 expression (450% increase compared to control) but LPS further enhanced the level of p62 in the presence of Baf inhibitor and again C16 failed to reverse the p62 increase. Interestingly, Aβ42 had no effect alone but significantly decreased (by 30%) p62 expression in the presence of Baf.

The co-labeling of p62, MAP2 for neurons, GFAP for astrocytes, and CD68 for microglia in the tri-culture showed that LPS causes accumulation of p62 specifically in microglia (Figure 2C for microglia and see Additional file 2 for neurons and astrocytes). *In situ* quantification of p62 fluorescence intensity showed that LPS increased by 184% for p62 compared to the control microglia. LPS-induced p62 increase in microglial cells was significantly higher than in neurons and astrocytes where p62 fluorescence intensity increased by 80% compared to control neurons, whereas LPS failed to significantly alter astrocytic p62 intensity (Figure 2D).

Concerning the conversion of LC3-I to LC3-II, the LC3-II/LC3-I ratio was calculated and represented in Figure 2B. As expected, blockade of the autophagic flux by Baf induced an accumulation of LC3-II; the LC3-II/LC3-I ratio was 5.45-fold of the control. Interestingly, the accumulation of LC3-II was more pronounced when cells were exposed to LPS in condition of blockade of the autophagic flux; LPS increased by 50% LC3-II/LC3-I ratio as compared to Baf alone. C16 failed to prevent this increase and Aβ42 had no effect.

Co-labeling of LC3, MAP2 for neurons, GFAP for astrocytes, and CD68 for microglia in the tri-culture (Figure 2E for microglia and see Additional file 2 for neurons and astrocytes) showed that, similarly to what was observed for p62, the largest LPS-induced increase in LC3 fluorescence intensity was observed in microglia and was significantly different from that quantified in neurons and astrocytes under LPS stress (56%, 116%, and 240% in neurons, astrocytes, and microglia compared to their respective control) (Figure 2F).

**Figure 3:**
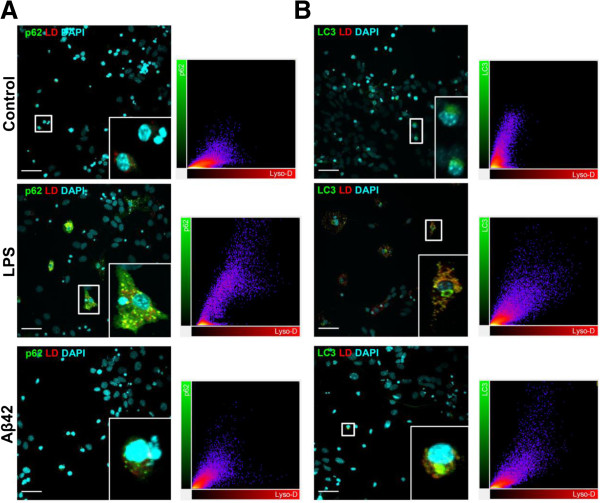
**Immunostaining of acidic vesicles and p62 or LC3 under inflammatory stress in primary tri-cultures.** Co-labeling of autophagic receptor **(A)** p62 (green) or **(B)** LC3 (green), Lyso-ID for acidic vesicles (red), and DAPI for nuclei (cyan) in tri-cultures seeded on coverslips, exposed to 20 μM Aβ42 or LPS 100 ng/mL in serum-free medium for 48 hours. All images were from a compilation of the entire z-series sections acquired by confocal microscopy (Olympus IX-81). A white square represents a magnified ROI. Two-dimensional fluorograms display the intensity and distribution of different colored pixels within the magnified ROI in the merged image. Scale bars, 42 μm. DAPI, 4′,6-diamidino-2-phenylindole; LPS, lipopolysaccharide; ROI, region of interest.

Using the Lyso-ID Red dye, an acidic organelle-selective dye, confocal images showed that many acidic vesicles were accumulated in tri-cultures treated with LPS, specifically in cells with microglial-like morphology (Figure 3). Merged images revealed that p62- and LC3-positive puncta largely co-localized with Lyso-ID-positive dots (Figure 3A,B, respectively).

Beclin-1 expression was not affected by LPS or Aβ42 treatments (data not shown).

### Activation of mTOR signaling pathway in primary tri-cultures

mTOR activation leads to phosphorylation of various substrates, in particular p70S6K at T389, a ribosomal S6 kinase involved in ribogenesis [26,27] and is also known as a negative regulator of autophagy; activation of mTOR leads to the inhibition of autophagy, whereas its inhibition by rapamycin activates autophagy.

**Figure 4:**
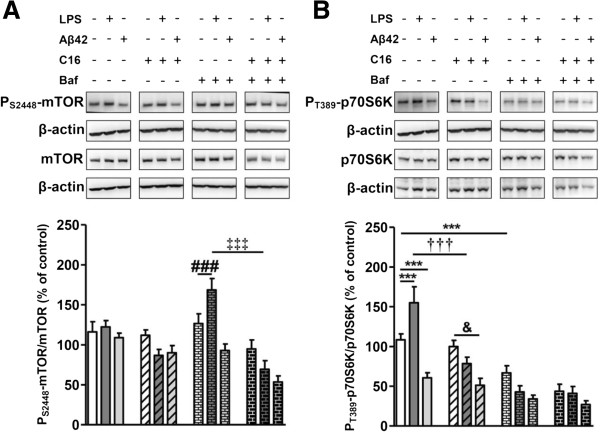
**Activation of mTOR signaling pathway by inflammatory stress in primary tri-cultures.** Representative immunoblots show the immunoreactivity of **(A)** mTOR, P_S2448_-mTOR, **(B)** p70S6K, P_T389_-p70S6K, and β-actin from cell lysates of primary tri-cultures exposed to 20 μM Aβ42 or 100 ng/mL LPS pretreated or not with 210 nM C16 in serum-free medium for 48 hours, treated or not with an autophagic flux inhibitor bafilomycin A1 (Baf) at 50 nM 24 hours before cell lysis. Densities were quantified using GeneTools software. Data of each protein were reported to data of the corresponding β-actin. The results are expressed as arbitrary units (percentage of the control set at 100%) and are mean ± SEM from six independent experiments in duplicate. ^***^P <0.001 compared to control; ^†††^*P* <0.001 compared to LPS; ^###^*P* <0.001 compared to Baf; ^‡‡‡^*P* <0.001 compared to LPS with Baf; ^&^*P* <0.05 compared to C16 by one-way ANOVA with a Newman-Keuls multiple comparison test. Baf, bafilomycin A1; LPS, lipopolysaccharide, mTOR, mammalian target of rapamycin.

Figure 4A shows that mTOR activation was only increased in the LPS with Baf condition (33% increase) which was significantly prevented by the addition of C16. Concerning p70S6K activation, LPS induced an increase in P_T389_-p70S6K/p70S6K (43%) which was prevented by C16, while Aβ42 decreased p70S6K activation (44%) which was maintained in the presence of C16. When the autophagic flux was blocked by Baf, p70S6K activation was inhibited (Figure 4B).

These results showed that: 1) only severe inflammatory stress induced by LPS led to an accumulation of acidic vesicles containing p62 and LC3 autophagic markers. Significant prevention of the rate of inflammatory factors by the C16 compound did not prevent the induction of autophagy; and 2) to our surprise, Aβ42 did not alter the rate of autophagic factors and did not induce inflammatory stress 48 hours after treatment compared to the control. We wanted to know whether an exogenous inflammatory stress in the presence of Aβ42 could alter autophagy by targeting three main cytokines, TNF-α, IL-1β, and IL-6, well-known in AD [15,28].

### Effect of exogenous inflammatory factors with Aβ42 in tri-cultures

#### Autophagy

Preliminary studies have shown that a cocktail of three cytokines (IL-1β, TNF-α, and IL-6) at doses ranging from 100 and 1,000 pg/mL in tri-cultures induced deleterious morphological changes starting at the dose of 400 pg/mL for 48 hours. Therefore, in the following experiments, the dose of 200 pg/mL was chosen because the cell integrity was preserved. In addition, the effects of each factor at a dose of 200 pg/mL on both inflammatory and autophagic components were determined in the presence or absence of 20 μM Aβ42.

As in the LPS condition, any change in Beclin-1 expression was observed by either the cocktail or individual inflammatory factors with or without Aβ42 or Baf (data not shown).

**Figure 5:**
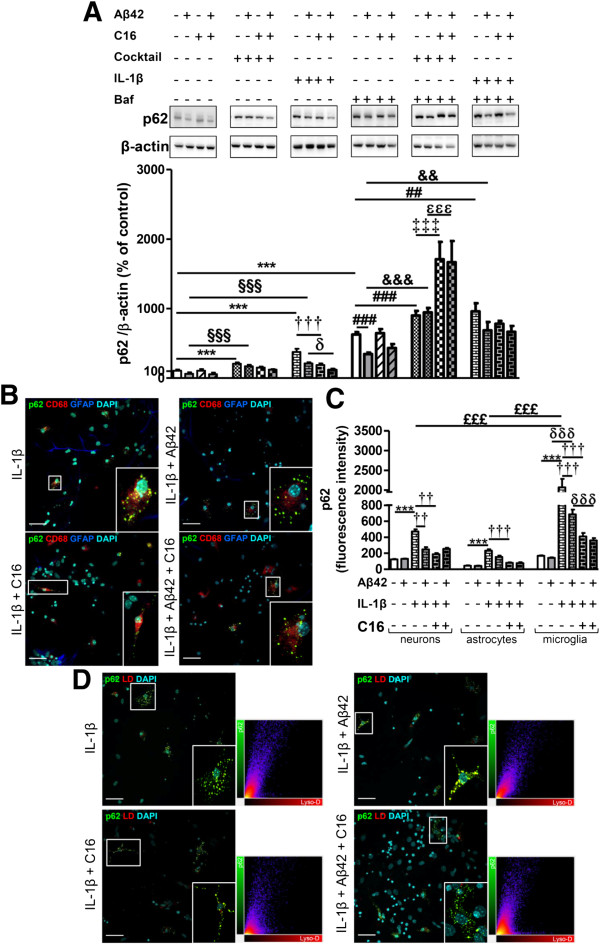
**Changes of p62 with cytokine cocktail or IL-1β stress in primary tri-cultures. (A)** Representative p62 and β-actin immunoblots from cell lysates of primary tri-cultures exposed or not to 20 μM Aβ42 pretreated or not with 210 nM C16 in serum-free medium and treated with a cytokine cocktail of 200 pg/mL of IL-1β, TNF-α, and IL-6 or with IL-1β alone for 48 hours, treated or not with an autophagic flux inhibitor bafilomycin A1 (Baf) at 50 nM 24 hours before cell lysis. Densities were quantified using GeneTools software. Data of each protein were reported to data of the corresponding β-actin. The results are expressed as arbitrary units (control set at 100%) and are mean ± SEM from six independent experiments in duplicate. **(B)** Co-labeling of the autophagic receptor p62 (green), CD68 for microglia (red), GFAP for astrocytes (blue), and DAPI for nuclei (cyan) in tri-cultures. **(C)** Fluorescence intensity of p62 staining in cells quantified using ImageJ software in confocal images. **(D)** Co-labeling of the autophagic receptor p62 (green), Lyso-ID (red), and DAPI for nuclei (cyan) in tri-cultures. In **(B)** and **(D)**, all images were from a compilation of the entire z-series sections acquired by confocal microscopy. A white square represents a magnified ROI. Two-dimensional fluorograms display the intensity and distribution of different colored pixels within the magnified ROI. Scale bars, 42 μm. ^***^P <0.001 compared to control; ^§§§^*P* <0.001 compared to Aβ42; ^††^*P* <0.01, ^†††^*P* <0.001 compared to IL-1β; ^δ^*P* <0.05, ^δδδ^*P* <0.001 compared to IL-1β with Aβ42; ^##^*P* <0.01, ^###^*P* <0.001 compared to Baf; ^&&^*P* <0.01, ^&&&^*P* <0.001 compared to Baf with Aβ42; ^‡‡‡^*P* <0.001 compared to Baf with cocktail; ^ϵϵϵ^*P* <0.001 compared to Baf with cocktail and Aβ42; ^£££^*P* <0.001 compared to IL-1β treatment in microglia by one-way ANOVA with a Newman-Keuls multiple comparison test. Baf, bafilomycin A1; DAPI, 4′,6-diamidino-2-phenylindole; GFAP, glial fibrillary acidic protein; ROI, region of interest.

In the absence of Baf, IL-1β and the inflammatory cocktail increased p62 by 94% and 253%, respectively, compared to the control. Furthermore, these inflammatory stresses applied with Aβ42 also increased the expression of p62 (60% and 98%, respectively), while Aβ42 alone had the tendency to decrease the level of expression of p62 (Figure 5A). Interestingly, C16 only prevented an IL-1β-induced increase in p62 with or without Aβ42. In the presence of Baf, the inflammatory cocktail and IL-1β enhanced the p62 expression with or without Aβ42 (Figure 5A) as it was observed for LPS in Figure 2A. However, the induction of inflammatory stress with TNF-α or IL-6 alone did not impair p62 expression [see Additional file 3A]. Consequently, confocal microscopy staining was only performed in cells treated with exogenous IL-1β and showed that microglia displayed significantly higher fluorescent p62 staining (12.4 times higher than the control microglia) compared to neurons and astrocytes (fluorescence intensity of 3.8 and 5.3 times higher than their respective controls) (Figure 5B,C). Furthermore, C16 treatment prevented the p62-positive staining in all cell types and, interestingly, p62 fluorescent intensity was also reduced by Aβ42 in microglia (Figure 5C). Accumulation of acidic vesicles stained by Lyso-ID and co-localized with p62 was prevented by C16 treatment in the IL-1β stress condition (Figure 5D).

**Figure 6:**
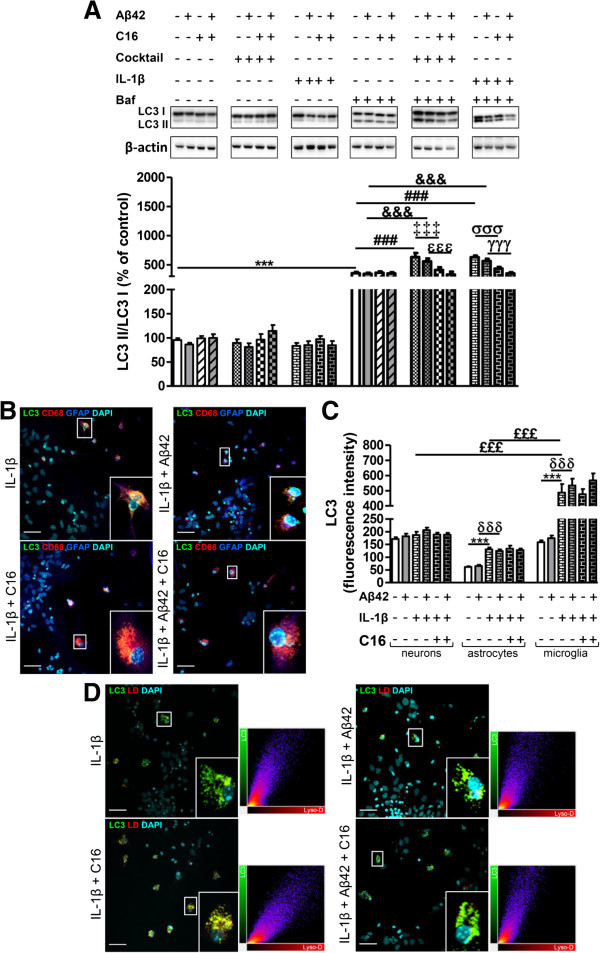
**Changes of LC3 with cytokine cocktail or IL-1β stress in primary tri-cultures. (A)** Representative LC3 and β-actin immunoblots from cell lysates of primary tri-cultures exposed or not to 20 μM Aβ42 pretreated or not with 210 nM C16 in serum-free medium and treated with a cytokine cocktail of 200 pg/mL of IL-1β, TNF-α, and IL-6 or with IL-1β alone for 48 hours, treated or not with an autophagic flux inhibitor bafilomycin A1 (Baf) at 50 nM for 24 hours before cell lysis. Densities were quantified using GeneTools software. Data of each protein were reported to data of the corresponding β-actin. The results are expressed as arbitrary units (control set at 100%) and are mean ± SEM from six independent experiments in duplicate. **(B)** Co-labeling of LC3 (green), CD68 for microglia (red), GFAP for astrocytes (blue), and DAPI for nuclei (cyan) in tri-cultures. **(C)** Fluorescence intensity of LC3 staining in neurons, astrocytes, and microglia quantified using ImageJ software in confocal images. **(D)** Co-labeling of LC3 (green), Lyso-ID (red), and DAPI for nuclei (cyan) in tri-cultures. In **(B)** and **(D)**, all images were from a compilation of the entire z-series sections acquired by confocal microscopy (Olympus IX-81). A white square represents a magnified ROI. Two-dimensional fluorograms display the intensity and distribution of different colored pixels within the magnified ROI in the merged image. Scale bars, 42 μm.^***^P <0.001 compared to control; ^δδδ^*P* <0.001, compared to IL-1β with Aβ42; ^###^*P* <0.001 compared to Baf; ^&&&^*P* <0.001 compared to Baf with Aβ42; ^‡‡‡^*P* <0.001 compared to Baf with cocktail; ^ϵϵϵ^*P* <0.001 compared to Baf with cocktail and Aβ42; ^σσσ^*P* <0.001 compared to Baf with IL-1β; ^γγγ^*P* <0.001 compared to Baf with IL-1β and Aβ42; ^£££^*P* <0.001 compared to IL-1β treatment in microglia by one-way ANOVA with a Newman-Keuls multiple comparison test. Baf, bafilomycin A1; DAPI, 4′,6-diamidino-2-phenylindole; GFAP, glial fibrillary acidic protein; ROI, region of interest.

Regarding LC3, western blot analysis showed that in the presence of Baf, inflammatory cocktail and IL-1β with or without Aβ42 increased the LC3-II/LC3-I ratio compared to Baf alone (Figure 6A). Contrary to LPS (Figure 2B), the compound C16 prevented these increases of the LC3-II/LC3-I ratio compared to Baf alone (Figure 6A). Similarly to what was observed for p62, TNF-α or IL-6 did not modify the LC3-II/LC3-I ratio with or without Aβ42 [see Additional file 3B]. LC3 immunostaining showed that under IL-1β stress, microglia displayed diffuse LC3 staining in the cytoplasm which was not prevented by C16 (Figure 6B). IL-1β induced more expression of LC3 in microglia than in astrocytes (207% and 113% increase of fluorescent intensity in microglia and astrocytes, respectively) (Figure 6C). Furthermore, co-labeling of LC3 and Lyso-ID showed that LC3 was found in many acidic vesicles under IL-1β stress with or without Aβ42 (Figure 6D).

Analysis of mTOR signaling showed that contrary to LPS, the inflammatory cocktail or each cytokine tested alone failed to activate mTOR [see Additional file 4A]. However, the inflammatory cocktail, TNF-α, and IL-6 activated p70S6K as shown for LPS and this activation was prevented by C16 only in the case of the inflammatory cocktail [see Additional file 4B]. In addition, Aβ42 significantly decreased p70S6K activation even in the presence of the inflammatory cocktail and cytokines TNF-α and IL-6 alone (between 45% and 60%). A decrease of P_T389_-p70S6K/p70S6K was also observed in the presence of IL-1β (67% and 64% with or without C16, respectively) [see Additional file 4B].

#### Inflammatory levels

**Figure 7:**
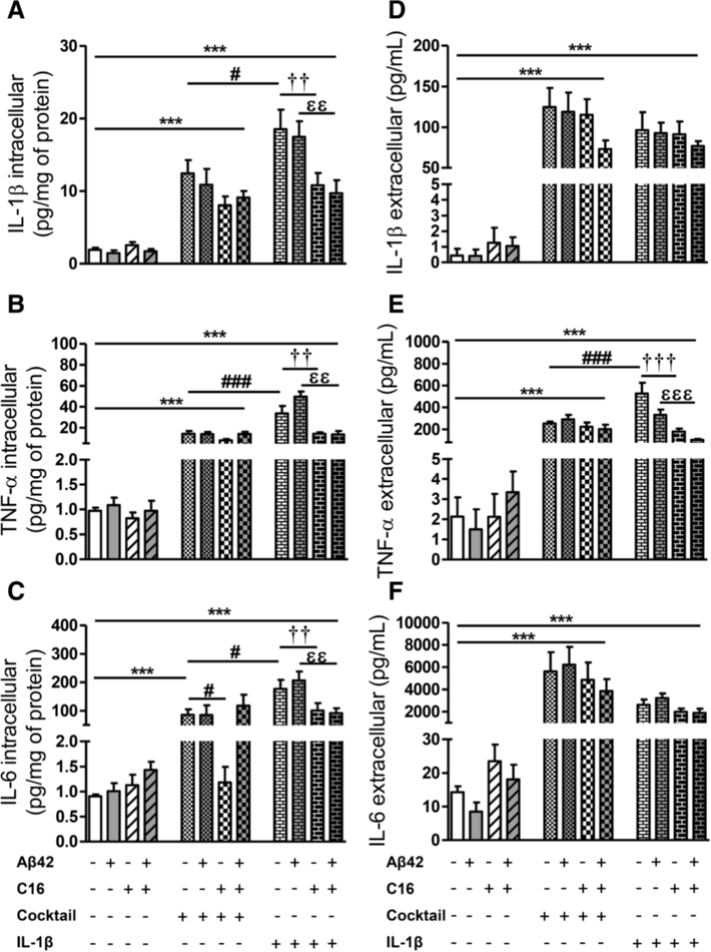
**Cytokine levels induced after an inflammatory cocktail or IL-1β stress in primary tri-cultures.** Cytokine (IL1-β, TNF-α, and IL-6) levels in cell lysates (**A**, **B**, **C**, respectively) and in culture medium (**D**, **E**, **F**, respectively) of tri-cultures pre-treated or not with 210 nM C16 and exposed or not to 20 μM Aβ42, and treated with a cocktail of IL-1β, TNF-α, and IL-6 (200 pg/mL of each cytokine) or with 200 pg/mL IL-1β alone in serum-free medium were analyzed by the 3-plex Luminex xMAP assay containing a mixture of specific beads for each cytokine as described in the Methods section. Cytokine levels in cell lysates and culture medium are expressed in pg/mg protein and pg/mL, respectively. Results are mean ± SEM for six experiments in duplicate. ^***^P <0.001 compared to respective control; ^#^*P* <0.05, ^###^*P* <0.001 compared to inflammatory cocktail; ^††^*P* <0.01, ^†††^*P* <0.001 compared to IL-1β, ^ϵϵ^*P* <0.01, ^ϵϵϵ^*P* <0.001 compared to IL-1β with Aβ42 by one-way ANOVA with a Newman–Keuls multiple comparison test.

The cytokine cocktail (200 pg/mL of IL-1β, TNF-α, and IL-6) and IL-1β (200 pg/mL) alone in tri-cultures of neurons/astrocytes/microglia induced a great increase of all cytokines in the intracellular compartment after 48 hours of treatment (Figure 7). Indeed, intracellular IL-1β levels were 3 to 8 times higher and 4 to 12 times higher than the control with cocktail and IL-1β treatment, respectively. While with cocktail, C16 had no effect, it significantly prevented the increase in the intracellular IL-1β induced by exogenous IL-1β with or without Aβ42 (Figure 7A). Intracellular TNF-α increases (about 12% with cocktail and 40% with IL-1β treatment) were observed and as for IL-1β, C16 only prevented the TNF-α production induced by IL-1β treatment (Figure 7B). Cocktail or IL-1β treatment induced an increase of intracellular IL-6 levels (90 and 200 times higher than the control, respectively). However, C16 prevented cocktail-induced production of IL-6 without Aβ42 and as for TNF-α and IL-1β, it inhibited the production of IL-6 induced by IL-1β treatment with or without Aβ42 (Figure 7C).

In the extracellular compartment, IL-1β levels with cocktail or IL-1β alone treatments were similar and lower than the dose treatment (mean: 96.68 ± 6.82 pg/mL) (Figure 7D). TNF-α levels induced by cocktail were similar to dose treatment (mean: 241.60 ± 19.50 pg/mL), while with IL-1β treatment, an increase was observed (*P* <0.001) without Aβ42 and compared to cocktail, and significantly prevented by C16 (66% and 69% in the absence or presence of Aβ42, respectively) (Figure 7E).

Extracellular IL-6 levels were higher than the amount included in exogenous cocktail (mean: 5143 ± 510 pg/mL versus 200 pg/mL in cocktail treatment) and a great release was also observed with IL-1β treatment (mean: 3643 ± 481 pg/mL) with no rescue by C16 (Figure 7F).

Concerning treatments of tri-cultures with TNF-α or IL-6 alone at 200 pg/mL, IL-1β and intracellular TNF-α and IL-6 levels were under the limit of detection. In the extracellular compartment, TNF-α treatment did not modify IL-6 levels, while IL-6 treatment induced a release of TNF-α (mean: 20.10 ± 2.97 pg/mL) but C16 had no effect [see Additional file 5].

This part of the results showed that: 1) a more moderate inflammation than previously induced by LPS also led to an accumulation of acidic vesicles containing LC3 and p62 even in the presence of Aβ42 in the tri-cultures; 2) this effect is ascribed only to IL-1β; 3) interestingly, the C16 compound prevented not only the production of intracellular factors TNF-α and IL-1β induced by exogenous IL-1β but also the accumulation of p62 and LC3 in acidic vesicles; and 4) again, we found that increases in p62 and in the conversion of LC3-I in LC3-II were more pronounced in microglia, where Aβ42 reversed the IL-1β-induced increase in p62, underlying an increased sensitivity of microglial autophagy to inflammatory stress in an integrated cellular environment modeling the brain parenchyma. We then wanted to confirm that the global changes of autophagy observed in tri-cultures mainly results from changes of microglial autophagy. Given the drastic alterations on neurons in these conditions, we investigated the impact of exogenous IL-1β with or without Aβ42 on purified cultures of astrocytes or microglia.

### Inflammation and autophagy in purified microglia and astrocyte cultures

#### Inflammatory level

**Figure 8:**
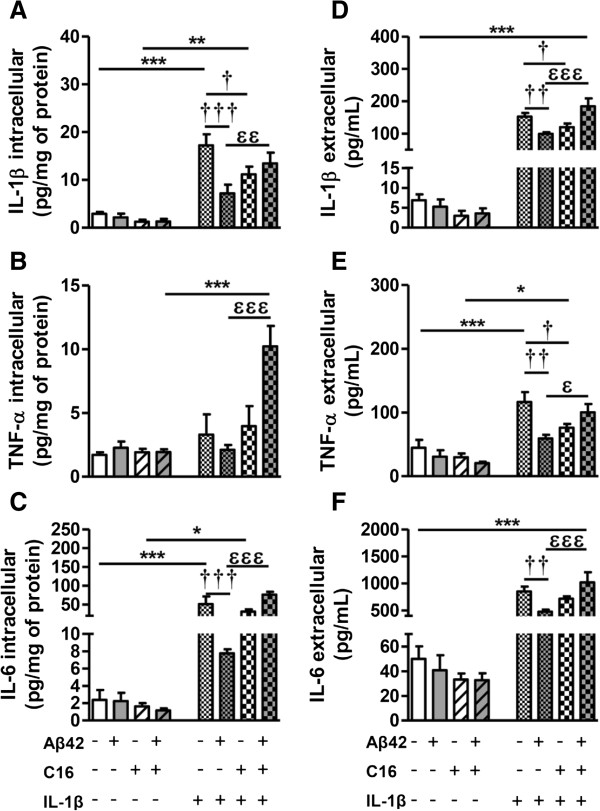
**Cytokine levels induced by inflammatory stress in microglia.** Cytokine (IL1-β, TNF-α, and IL-6) levels in cell lysates (**A**, **B**, **C**, respectively) and in culture medium (**D**, **E**, **F**, respectively) of primary microglia pre-treated or not with 210 nM C16, exposed or not to 20 μM Aβ42, and treated with 200 pg/mL of IL-1β in serum-free medium were analyzed by the 3-plex Luminex xMAP assay containing a mixture of specific beads for each cytokine as described in the Methods section. Cytokine levels in cell lysates and culture medium are expressed in pg/mg protein and pg/mL, respectively. Results are mean ± SEM for six experiments in duplicate. ^*^P <0.05, ^**^P <0.01, ^***^P <0.001 compared to respective control; ^†^*P* <0.05, ^††^*P* <0.01, ^†††^*P* <0.01 compared to IL-1β; ^ϵ^*P* <0.05, ^ϵϵ^*P* <0.01, ^ϵϵϵ^*P* <0.001 compared to Aβ42 with IL-1β by one-way ANOVA with a Newman–Keuls multiple comparison test.

Treatment of microglia with 200 pg/mL IL-1β for 48 hours induced a great increase of intracellular cytokines. In fact, IL-1β levels were 6 to 10 times higher than respective controls except in the presence of Aβ42 where levels were similar to Aβ42 alone (Figure 8A). The C16 treatment partially prevented IL-1β production (35%, *P* <0.05) induced by exogenous IL-1β without Aβ42. However, C16 reversed the inhibitory Aβ42 effect on IL-1β production (Figure 8A). For TNF-α, exogenous IL-1β only increased intracellular levels in the presence of Aβ42 and C16 (Figure 8B). For IL-6, intracellular levels increased by a factor of 20 to 66 compared to respective controls except in Aβ42 with IL-1β treatment where they were similar to Aβ42 alone (Figure 8C). The C16 treatment did not prevent IL-6 production induced by exogenous IL-1β without Aβ42. However, C16 blocked the Aβ42 inhibitory effect on IL-6 production (Figure 8C).

In the extracellular compartment, results showed that Aβ42 decreased IL-1β levels (35%, *P* <0.01). The C16 treatment also decreased IL-1β levels (21%, *P* <0.05) without Aβ42. However, in the presence of Aβ42, C16 blocked the effect of Aβ42; extracellular IL-1β levels were similar to the dose treatment (Figure 8D).

Exogenous IL-1β induced a great increase of extracellular levels of TNF-α (mean: 108.40 ± 8.10 pg/mL for IL-1β alone or with both Aβ42 and C16). However, Aβ42 decreased TNF-α levels induced by IL-1β treatment (49%, *P* <0.01). The C16 also decreased TNF-α levels induced by IL-1β treatment (35%, *P* <0.05). However, in the presence of Aβ42, C16 blocked the effect of Aβ42 (Figure 8E). As for TNF-α, exogenous IL-1β significantly increased extracellular levels of IL-6 in all conditions (mean: 766.40 ± 114.60 pg/mL) (Figure 8F). However, Aβ42 decreased IL-6 levels induced by IL-1β treatment (44%, *P* <0.01). C16 blocked the inhibitory effect of Aβ42 (Figure 8F).

In astrocytes, IL-1β treatment did not induce production of IL-1β [see Additional file 6A] and in the extracellular compartment, levels were significantly decreased compared to the dose added in the medium (mean: 43.17 ± 4.42 pg/mL versus 200 pg/mL). In the presence of C16, results showed an increase in extracellular IL-1β levels (36% and 50%, without and with Aβ42, respectively) [see Additional file 6C]. IL-1β induced IL-6 production, which was higher in the presence of C16, but IL-6 levels remained low at less than 10 pg/mL [see Additional file 6B]. In the extracellular compartment, results were similar to those of IL-1β, with a significant increase in the presence of C16 (100% and 176%, without and with Aβ42, respectively) [see Additional file 6D]. For TNF-α, levels were under the limit of detection.

#### Autophagy monitoring in microglia and astrocytes

**Figure 9:**
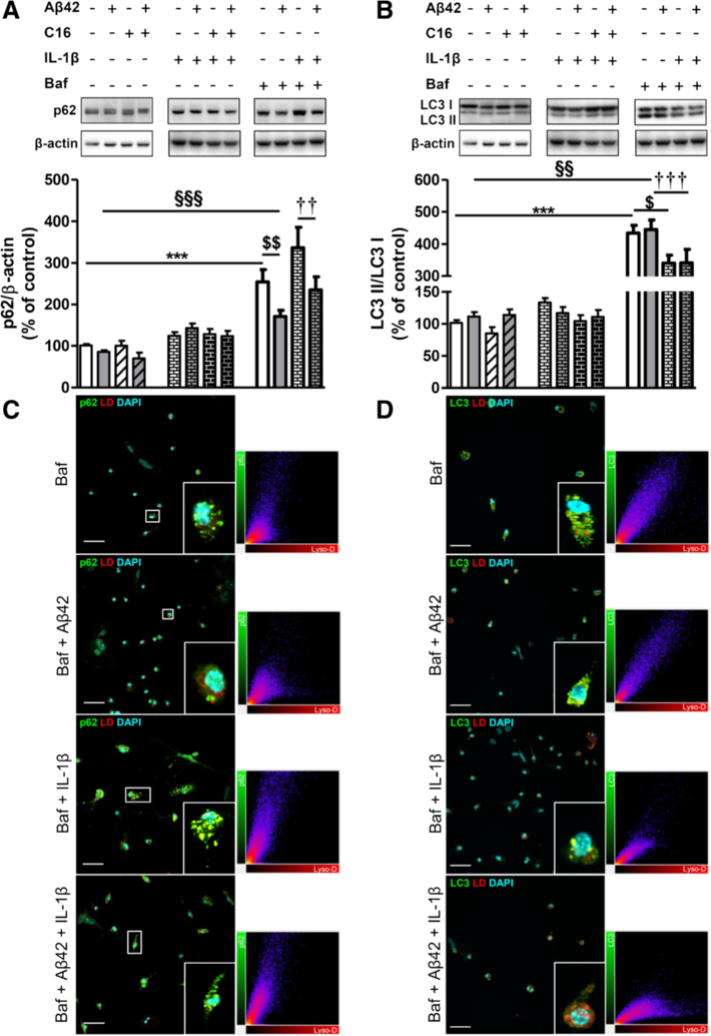
**Changes in autophagic factors in microglia.** Representative immunoblots show the immunoreactivity of **(A)** p62, (**B)** LC3-I, LC3-II, and β-actin from cell lysates of primary microglia pretreated or not with 210 nM C16 and exposed or not to 20 μM Aβ42 in serum-free medium containing or not 200 pg/mL of IL-1β for 48 hours. The autophagic flux inhibitor bafilomycin A1 (Baf) at 50 nM was added or not for 24 hours before cell lysis. Densities were quantified using GeneTools software. Data of each protein were reported to data of the corresponding β-actin. The results are expressed as arbitrary units (percentage of the control set at 100%) and are mean ± SEM from six independent experiments in duplicate. ^***^P <0.001 compared to control; ^§§^*P* <0.01, ^§§§^*P* <0.001 compared to Aβ42; ^$^*P* <0.05, ^$$^*P* <0.01 compared to Baf; ^††^*P* <0.01, ^†††^*P* <0.001 compared to Aβ42 with IL-1β and Baf by one-way ANOVA with a Newman-Keuls multiple comparison test. Co-labeling of the autophagic receptor **(C)** p62 (green) or **(D)** LC3 (green), Lyso-ID (red), and DAPI for nuclei (cyan) in microglia. All images were from a compilation of the entire z-series sections acquired by confocal microscopy (Olympus IX-81). For **(A)** and **(B)**, only merged images were selected. A white square represents a magnified ROI. Two-dimensional fluorograms display the intensity and distribution of different colored pixels within the magnified ROI in the merged image. Scale bars, 42 μm. Baf, bafilomycin A1; DAPI, 4′,6-diamidino-2-phenylindole; ROI, region of interest.

Concerning p62 in microglial cell cultures, treatment with IL-1β alone or combined with Aβ42 increased p62 by 30%, whereas Aβ42 alone had no effect. P62 increased by 92% and 173% with Baf alone and IL-1β plus Baf, respectively (Figure 9A). Aβ42 partially reversed the Baf-induced increase of p62 (40%, *P* <0.001) and significantly prevented the p62 increase induced by IL-1β combined with Baf (37%, *P* <0.001) (Figure 9A).

Regarding the LC3-II/LC3-I ratio, Aβ42 and IL-1β alone or combined had no significant effect without Baf. However, in the presence of Baf, the LC3-II/LC3-I ratio increased by 329% and 300% for Baf alone and Baf with Aβ42, respectively (Figure 9B). Microglial cell treatment with IL-1β in the presence of Baf significantly reduced the LC3-II/LC3-I ratio by 22% and 28% compared to Baf alone or Baf plus Aβ42, respectively (Figure 9C).

Similarly to what was observed in tri-cultures, immunofluorescence analysis showed that p62 increased in microglia exposed to IL-1β with or without Aβ42. P62 was largely found within acidic vesicles stained with Lyso-ID [see Additional file 7A]. In the presence of Baf autophagy inhibitor, the accumulation of p62 co-localized with Lyso-ID was even more pronounced and, interestingly, was prevented by Aβ42 with or without IL-1β (Figure 9C). While no difference was observed in LC3 expression without Baf (Figure 9B and see Additional file 7B), co-immunostaining of LC3 and Lyso-ID was positive in the presence of Baf with or without Aβ42 which was prevented by the IL-1β treatment (Figure 9D).

The inhibitory effect of Aβ42 on the production of inflammatory factors and accumulation of acid vesicles containing p62 induced by IL-1β was not an effect on cell death because the result of the MTS test (Promega, Charbonnières les Bains, France) showed no difference compared to the control microglia (100.00 ± 4.00% and 103.60 ± 2.86% cell viability for control microglia and Aβ42-treated microglia, respectively; n = 6 experiments in duplicate).

**Figure 10:**
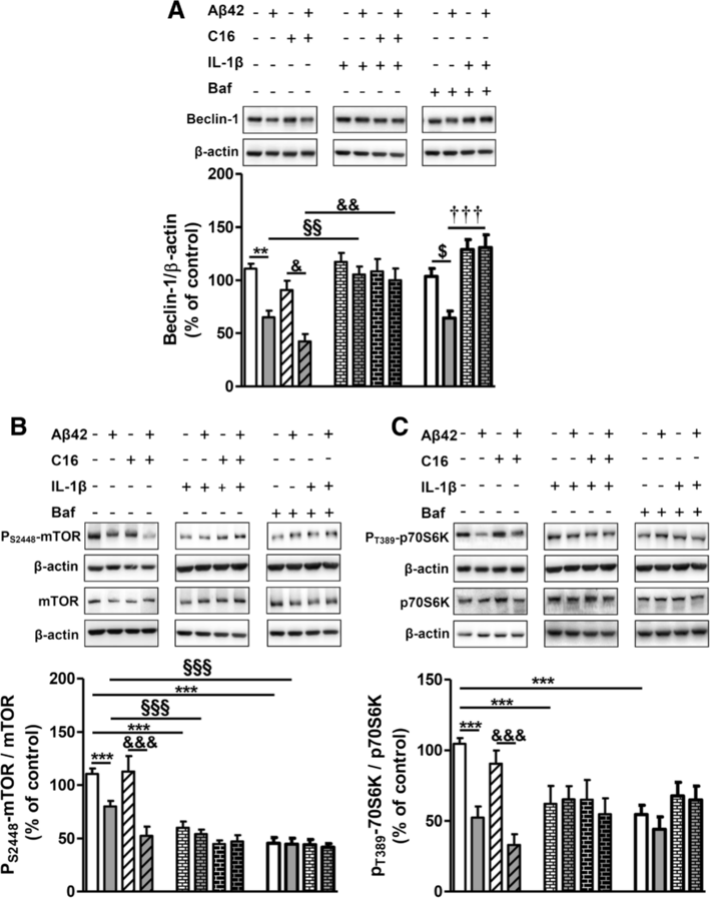
**Activation of mTOR signaling pathway in microglia.** Representative immunoblots show the immunoreactivity of **(A)** Beclin-1, **(B)** mTOR, **(C)** P_S2448_-mTOR, p70S6K, P_T389_-p70S6K, and β-actin from cell lysates of primary microglia pretreated or not with 210 nM C16 and exposed or not to 20 μM Aβ42 in serum-free medium and treated with 200 pg/mL of IL-1β for 48 hours, treated or not with an autophagic flux inhibitor bafilomycin A1 (Baf) at 50 nM for 24 hours before cell lysis. Densities were quantified using GeneTools software. Data of each protein were reported to data of the corresponding β-actin. The results are expressed as arbitrary units (percentage of the control set at 100%) and are mean ± SEM from six independent experiments in duplicate. ^***^P <0.001 compared to control; ^§§§^*P* <0.001 compared to Aβ42; ^&&&^*P* <0.001 compared to C16 by one-way ANOVA with a Newman-Keuls multiple comparison test. Baf, bafilomycin A1; mTOR, mammalian target of rapamycin.

In microglia, Aβ42 treatment induced a decrease of Beclin-1 of 42% and 54% without or with C16 pre-treatment, respectively, which was suppressed by IL-1β treatment (Figure 10A). As in tri-cultures, Aβ42 decreased the expression of Beclin-1 in the Baf condition which was also inhibited by IL-1β treatment (Figure 10A).

Contrary to tri-cultures, Aβ42 and IL-1β alone or combined significantly decreased the mTOR activation which was not prevented by C16 pre-treatment in microglia (between 30% and 50%) (Figure 10B). Furthermore, these inhibitions were associated with a great decrease of P_T389_-p70S6K/p70S6K ratio (between 40% and 60%) as shown in Figure 10C. As in tri-cultures, Baf decreased both mTOR and its downstream substrate p70S6K activations (Figure 10B,C).

In astrocytes no changes in autophagic factors were observed except in the presence of Baf where all of them were increased [see Additional file 8A,B,C]. Aβ42 and IL-1β alone or in association significantly inhibited the mTOR signaling pathway not prevented by C16 [see Additional file 8D,E].

The latest results obtained in purified microglia showed that: 1) exogenous IL-1β induced p62 accumulation in acidic vesicles and production of IL-1β and TNF-α which were significantly prevented by Aβ42, suggesting that amyloid peptide could maintain microglia defense; 2) the C16 compound inhibited the effects of Aβ42, indicating that its inhibitory role on PKR activation could be dangerous for microglial autophagy (see Discussion section); and 3) contrary to microglia, exogenous IL-1β did not induce autophagy in purified astrocytes.

## Discussion

Different studies have demonstrated a close relationship between inflammation and autophagy in Crohn’s disease [29], cancer [30], cutaneous inflammation [31], and diabetes [32]. Inflammation also constitutes a key component in the pathogenesis of AD. Therefore, multiple anti-inflammatory treatments have been tested but they were not satisfactory [33]. Furthermore, autophagy has been shown to be impaired in AD with accumulation of AVs containing proteins for Aβ production [8,9]. For the first time, this study brought out the links between autophagy and inflammation in AD using a primary tri-culture modeling the brain parenchyma by including neurons, astrocytes, and microglia as previously described [17].

We first treated tri-cultures with LPS, known to induce autophagy and to produce cytokines through Toll-like receptor (TLR)-4 activation [34,35]. Interestingly, under this robust inflammatory input, microglia was very reactive with many p62- and LC3-positive puncta in cytoplasm and ramified processes, suggesting autophagy induction specifically in microglia, while neurons were condensed with very short extensions or died and many astrocytes were stellar without p62 and LC3 signals. Moreover, this immunostaining was predominantly co-localized with the Lyso-ID Red dye staining acidic vesicles. TLR is well known as a major innate immune sensor and has been shown to mediate autophagy through the recruitment of different protein adaptors such as p62 [36]. However, the LC3-II/LC3-I ratio was not changed compared to the control, except in the presence of bafilomycin, an inhibitor of autophagy, indicating that LPS induces autophagy in our experimental settings, confirming previous findings.

Unexpectedly, treatment of tri-cultures with Aβ42 did not affect the expression of p62 and the LC3-II/LC3-I ratio, except in the presence of Baf where p62 expression significantly decreased. Aβ42 alone did not modify autophagy in the serum-free conditions where a basal inflammation was similar to the control after 48 hours of treatment. Other studies showed that Aβ neurotoxicity induced cytokine mRNA expression [37,38] but few data are available concerning TNFα, IL-1β, and IL-6 by ELISA after Aβ42 treatment *in vitro*. Meda *et al*. [39] indicated that production of TNFα in Aβ25-35-treated microglia was only observed after stimulation by IFN-γ. Other studies showed in microglia that oligomers were only inducers of inflammatory factors but not the fibrillar form of amyloid peptide [40]. Furthermore, in human fetal microglia, it was shown that Aβ42 induced release of TNFα and IL-1β but levels were around 10 and 8 pg/mL, respectively, accordingly to our results [41]. These last authors also showed that the production was enhanced by IL-8.

To gain a better understanding of the autophagic process, we checked the mTOR signaling pathway. In our conditions, the mTOR activation was similar with LPS or Aβ42, except with Baf where LPS increased the mTOR activation. The p70S6K activation was increased by LPS and conversely decreased with Aβ42 as previously described [42,43].

Taken together, LPS, a robust inflammatory inducer, activated the mTOR/p70S6K pathway and induced autophagy with accumulation of many acidic p62- and LC3-positive vesicles in our experimental conditions. Many studies support this LPS toxicity [44-48]. However, Aβ42, which induced a low grade of inflammation, inhibited the activation of the mTOR pathway and could activate autophagy. For the first time, we described the role of Aβ42 on the autophagic flux in primary neurons, astrocytes, and microglia. Only one *in vitro* study has examined the autophagy using SH-SY5Y cells exposed to Aβ42 and showed autophagosome accumulation [49]. Besides, many authors have shown AVs in transgenic mouse models but the inflammation is severe in mice and often studied at a very advanced age [49-52]. Thus, Aβ42 alone could stimulate autophagy at early stages of the disease and progressively a deleterious flux of autophagy could take place with inflammatory input.

The C16 compound known for its anti-inflammatory properties via the PKR inhibition [17,20,53] prevents at least 50% of the production and release factors IL-1β, TNF-α, and IL-6 (except its release) induced by LPS. However, this effect while reducing the LPS-induced activation of mTOR/p70S6K did not rescue the induction of autophagy. It is clear that mTOR is involved in innate and adaptive immunity [54,55]. Indeed, the mTOR activation induced cytokine production, which in turn can also activate mTOR [56,57]. C16 by inhibiting cytokine production can inhibit the mTOR pathway. Thus, the robust inflammation by LPS could alter autophagy independently of mTOR. Furthermore, C16 did not prevent IL-6 release induced by LPS, and recently it was shown that this cytokine can promote the maturation of autophagosomes by regulating extracellular signal-regulated kinase (ERK) which is not a target of C16 [58] and IL-6 can inhibit autophagy through interaction of STAT3 with PKR [59].

In a second part, as Aβ42 did not modify autophagy in tri-cultures contrary to LPS and in order to better understand cytokine involvement in autophagy regulation in AD, we tested the impact of a cytokine cocktail (IL-1β, TNF-α, and IL-6) and each cytokine alone in tri-cultures exposed to Aβ42. Some studies demonstrated that cytokines such as TNF-α, IL-1β, and IL-6 can induce autophagy in infected macrophages as a defense mechanism [60]. However, only a cytokine cocktail or IL-1β increased the p62 expression and LC3-II/LC3-I ratio (with Baf) as LPS with or without Aβ42. By co-immunostaining, tri-cultures treated with cocktail or IL-1β displayed p62- and LC3-positive puncta co-localized with acidic vesicles in reactive microglia as in LPS. Contrary to the cocktail, the autophagic changes observed with exogenous IL-1β were rescued by C16 which partially inhibited all cytokine production induced by the exogenous IL-1β treatment. By inhibiting the PKR pathway, C16 could prevent inflammasome formation. Indeed, PKR physically interacts with inflammasome components, leading to inflammasome activation which produces IL-1β from pro-IL-1β by caspase-1 cleavage [21]. Furthermore, autophagy is a novel evolutionarily conserved function of the PKR pathway that is targeted by viral virulence gene products [61,62] and in cancer where anti-tumor effects induce autophagosome formation through PKR activation [63].

Contrary to LPS and the cytokine cocktail, IL-1β inhibited the p70S6K activation as Aβ42 that would support a compensatory stimulation of autophagy. However, IL-1β also showed an accumulation of acidic vesicles loaded with p62 and LC3 unlike Aβ42 alone.

These findings suggest that IL-1β could be a critical inducer of autophagy dependent on the PKR activation and associated to a great accumulation of acidic vesicles with or without Aβ42, suggesting a regulation of autophagy by IL-1β with the progression of the disease. However, IL-1β has a controversial role in AD depending on experimental models. Indeed, some authors demonstrated that IL-1β overexpression decreased amyloid deposits in inducible APP/PS1-IL-1β^XAT^ or 3xTgAD-IL-1β^XAT^ mice receiving a unilateral stereotactic injection of FIV-Cre, but exacerbated tau pathology [64,65]. They also observed infiltrating inflammatory neutrophils, suggesting an involvement of peripheral cells to eliminate amyloid deposits as previously described [66]. On the contrary, blocking IL-1β decreased pro-inflammatory cytokines, enhanced microglial phagocytosis of Aβ, and rescued cognition and tau pathology in 3xTgAD treated with intraperitoneal anti-IL-1 receptor [67]. Recently, APP/PS1 mice with NLRP3 inflammasome deficiency were largely protected from the loss of spatial memory and resulted in decreased Aβ deposits [16]. In many AD transgenic mice, autophagic flux was inhibited with accumulation of AVs, but it is well established that autophagy regulates IL-1β secretion [6] by degradation of inflammasome [68,69]. Thus, a therapeutic strategy aiming to enhance autophagy in AD could be attractive to limit the deleterious effects of IL-1β.

Interestingly, accumulation of acidic p62^+^ and LC3^+^ vesicles by LPS, cytokine cocktail, or IL-1β was observed in cells that looked like microglia. In a third part, to confirm, we investigated the IL-1β treatment in purified microglia and astrocytes. For these last cells, any change was observed in the p62 expression and LC3-II/LC3-I ratio, while the mTOR/p70S6K activation decreased with or without Aβ42. While the role of astrocytes is increasingly appreciated [70], their autophagic process has never been studied in AD. In severe lysosomal storage disorders, Di Malta *et al*. [71] showed that autophagy in astrocytes was impaired.

In purified microglia cultures, the IL1-β treatment induced the production of cytokines prevented by Aβ42 in the absence of C16. As the mTOR pathway was inhibited with both Aβ42 and IL1-β, the decrease of IL1-β-induced cytokine production by Aβ42 could not be explained by alteration of protein synthesis. In addition, no microglia death was observed with Aβ42. This cytokine inhibition by Aβ42 was lost in the presence of the PKR inhibitor, indicating the involvement of this kinase in the cytokine production in microglia. Aβ42 by activating PKR could induce a defense reaction of microglia as non-viral pathogens which induced autophagy by PKR activation [72]. Thus, in microglia, it could be proposed that a PKR-dependent autophagy could be playing a positive role to limit IL-1β toxicity. In microglia, Aβ42 decreased Beclin-1 and p62 without modification of the LC3-II/LC3-I ratio. Interestingly, Lyso-ID Red vesicles were less loaded with autophagic markers than with IL1-β, suggesting no impairment of autophagic flux in our experimental conditions. These findings were in accordance with data that showed that active autophagy reduced IL1-β production [5] and inflammasome deficiency in AD mouse models limited Aβ deposits and improved microglial phagocytosis [16].

It should be noted that these results in purified microglia are not completely congruent with those in tri-cultures. The microglia was more amoeboid with less p62 expression and decreased LC3-II/LC3-I ratio than in the tri-cultures where changes in autophagic factors were more sustained in microglia and extended many ramified processes. An increasing body of evidence suggests that neurons, astrocytes, and microglia cooperation influence inflammatory environment and their own activation [73-75].

## Conclusion

These results highlight that IL-1β induced autophagy with accumulation of many acidic vesicles loaded with p62 and LC3 in microglia of tri-cultures and purified microglia. Interestingly, Aβ42 maintains autophagy in microglia and prevents effects of exogenous IL-1β in the production of inflammatory factors and in the autophagy impairment. In microglia, Aβ42 could generate an optimal host immune response through an active PKR-dependent autophagy.

Therefore, a better understanding of IL-1β levels and autophagy status in AD brains according to the stage of the disease would allow improved targeting of anti-IL-1β and pro-autophagic therapies to reduce cognitive decline.

## Abbreviations

Aβ42: Amyloid peptide; AD: Alzheimer’s disease; AMPK: AMP-activated protein kinase; ANOVA: Analysis of variance; Atg16L1: Autophagy-related protein 16-1; APP: Amyloid precursor protein; Atg16L1: Autophagy-related protein 16-1; AV: Autophagic vacuole; Aβ: Amyloid-beta; Baf: Bafilomycin A1; BSA: Bovine serum albumin; CNS: Central nervous system; DAPI: 4′,6-diamidino-2-phenylindole; DMEM: Dulbecco’s modified Eagle’s medium; DMSO: Dimethyl sulfoxide; DTT: Dithiothreitol; EDTA: Ethylenediaminetetraacetic acid; ELISA: Enzyme-linked immunosorbent assay; ERK: Extracellular signal-regulated kinase; FBS: Fetal bovine serum; FITC: Fluorescein isothiocyanate; GFAP: Glial fibrillary acidic protein; HRP: Horseradish peroxidase; IFN: Interferon; IgG: Immunoglobulin G; IL: Interleukin; LDS: Lithium dodecyl sulfate; LPS: Lipopolysaccharide; MAP2: Microtubule-associated protein 2; MEM: Minimum essential medium Eagle; MFI: Median fluorescence intensity; mTOR: Mammalian target of rapamycin; NaF: Sodium fluoride; NF-κB: Nuclear factor kappa-light-chain-enhancer of activated B cells; PBS: Phosphate buffer saline; PFA: Paraformaldehyde; PI3K: Phosphatidylinositide 3-kinase; PKR: Double-stranded RNA-dependent protein kinase; PLL: Poly-L-lysine; PMSF: Phenylmethylsulfonyl fluoride; PS: Penicillin-streptomycin; PS1: Presenilin 1; ROI: Region of interest; RPE: R-phycoerythrin; SDS: Sodium dodecyl sulfate; SEM: Standard error of the mean; STAT3: Signal transducer and activator of transcription 3; TBST: Tris-buffered saline and Tween; TLR: Toll-like receptor; TNF: Tumor necrosis factor; TRITC: Tetramethylrhodamine isothiocyanate.

## Competing interests

The authors declare that they have no competing interests.

## Authors’ contributions

GP and FT designed the research; AF and GP performed the research; AF, GP, and FT wrote the manuscript; AF analyzed the data; and ARB, TJ, and MP followed the study with constructive comments. All authors read and approved the final manuscript.

## Supplementary Material

Additional file 1 **Cytokine levels induced by inflammatory stress in autophagy inhibitory condition.** Cytokine (IL1-β, TNF-α and IL-6) levels in culture medium (**A**, **B**, **C**, respectively) and in cell lysates (**D**, **E**, **F**, respectively) of tri-cultures in serum-free medium treated with an autophagic flux inhibitor, 50 nM bafilomycin A1 (Baf), with 100 ng/mL LPS or with 20 μM Aβ42 and pre-treated or not with 210 nM C16, were analyzed by the 3-plex Luminex xMAP assay containing a mixture of specific beads for each cytokine as described in the Methods section. Cytokine levels in culture medium and cell lysates are expressed in pg/mL and pg/mg protein, respectively. Results are mean ± SEM for six experiments in duplicate. ^***^P <0.001 compared to Baf alone; ^###^*P* <0.001 compared to LPS with Baf; ^†^*P* <0.05, ^††^*P* <0.01, ^†††^*P* <0.001 compared to Baf with C16 by one-way ANOVA with a Newman-Keuls multiple comparison test. Baf, bafilomycin A1; LPS, lipopolysaccharide. Click here for file

Additional file 2 **Immunofluorescence of p62 and LC3 in neurons and astrocytes under inflammatory stress in primary tri-cultures.** Co-labeling of autophagic factor **(A)** p62 (red) or **(B)** LC3 (red), MAP2 for neurons (green), GFAP for astrocytes (blue), and DAPI for nuclei (cyan) in tri-cultures seeded on coverslips and exposed to 20 μM Aβ42 or LPS 100 ng/mL in serum-free medium for 48 hours. All images were from a compilation of the entire z-series sections acquired by confocal microscopy (Olympus IX-81). A white square represents a magnified ROI. Scale bars, 42 μm. DAPI, 4′,6-diamidino-2-phenylindole; GFAP, glial fibrillary acidic protein; LPS, lipopolysaccharide; MAP2, microtubule-associated protein 2; ROI, region of interest. Click here for file

Additional file 3 **Changes in autophagic factors under inflammatory stress.** Representative immunoblots show the immunoreactivity of **(A)** p62, **(B)** LC3-I, LC3-II, and β-actin from cell lysates of primary tri-cultures exposed or not to 20 μM Aβ42 pretreated or not with 210 nM C16 in serum-free medium and treated with 200 pg/mL of TNF-α or IL-6 alone for 48 hours, treated or not with an autophagic flux inhibitor bafilomycin A1 (Baf) at 50 nM for 24 hours before cell lysis. Densities were quantified using GeneTools software. Data of each protein were reported to data of the corresponding β-actin. The results are expressed as arbitrary units (percentage of the control set at 100%) and are mean ± SEM from six independent experiments in duplicate. ^***^P <0.001 compared to control; ^###^*P* <0.001 compared to Baf by one-way ANOVA with a Newman-Keuls multiple comparison test. Baf, bafilomycin A1. Click here for file

Additional file 4 **Activation of mTOR signaling pathway by exogenous cytokines in tri-cultures.** Representative immunoblots show the immunoreactivity of **(A)** mTOR, P_S2448_-mTOR, **(B)** p70S6K, P_T389_-p70S6K, and β-actin from cell lysates of primary tri-cultures exposed to 20 μM Aβ42, pretreated or not with 210 nM C16 in serum-free medium and treated with a cytokine cocktail of 200 pg/mL of IL-1β, TNF-α, and IL-6 or with 200 pg/mL of IL-1β, TNF-α, or IL-6 alone for 48 hours. Densities were quantified using GeneTools software. Data of each protein were reported to data of the corresponding β-actin. The results are expressed as arbitrary units (percentage of the control set at 100%) and are mean ± SEM from six independent experiments in duplicate. ^***^P <0.001 compared to control; ^&&^*P* <0.01, ^&&&^*P* <0.001 compared to C16; ^‡‡^*P* <0.01, ^‡‡‡^*P* <0.001 compared to inflammatory cocktail; ^$^*P* <0.05 compared to TNF-α; ^§§§^*P* <0.001 compared to TNF-α with C16; ^†^*P* <0.05 compared to IL-6; ^δ^*P* <0.05 compared to IL-6 with C16 by one-way ANOVA with a Newman-Keuls multiple comparison test. mTOR, mammalian target of rapamycin. Click here for file

Additional file 5 **Cytokine levels induced by exogenous TNF-α and IL-6.** Cytokine (TNF-α and IL-6) levels in culture medium (**A** and **B**, respectively) of tri-cultures pre-treated or not with 210 nM C16, exposed or not to 20 μM Aβ42, and treated with 200 pg/mL of TNF-α or IL-6 alone in serum-free medium were analyzed by the 3-plex Luminex xMAP assay containing a mixture of specific beads for each cytokine as described in the Methods section. Cytokine levels are expressed in pg/mL. Results are mean ± SEM for six experiments in duplicate. ^**^P <0.01, ^***^P <0.001 compared to respective control by one-way ANOVA with a Newman-Keuls multiple comparison test. Click here for file

Additional file 6 **Cytokine levels induced by inflammatory stress in astrocytes.** Cytokine (IL-1β and IL-6) levels in cell lysates (**A** and **B**, respectively) and in culture medium (**C** and **D**, respectively) of primary astrocytes pre-treated or not with 210 nM C16, exposed or not to 20 μM Aβ42, and treated with 200 pg/mL of IL-1β alone in serum-free medium were analyzed by the 3-plex Luminex xMAP assay containing a mixture of specific beads for each cytokine as described in the Methods section. Cytokine levels in cell lysates and culture medium are expressed in pg/mg protein and pg/mL, respectively. Results are mean ± SEM for six experiments in duplicate. ^***^P <0.001 compared to respective control; ^††^*P* <0.01, ^†††^*P* <0.001, compared to IL-1β; ^ϵϵ^*P* <0.01, ^ϵϵϵ^*P* <0.001 compared to Aβ42 with IL-1β by one-way ANOVA with a Newman-Keuls multiple comparison test. Click here for file

Additional file 7 **Immunostaining of p62 or LC3 and Lyso-ID in purified microglia.** Co-labeling of the autophagic receptor **(A)** p62 (green) or **(B)** LC3 (green), Lyso-ID (red), and DAPI for nuclei (cyan) in microglia seeded on coverslips and exposed to 20 μM Aβ42 or 200 pg/mL IL-1β in serum-free medium for 48 hours. All images were from a compilation of the entire z-series sections acquired by confocal microscopy (Olympus IX-81). A white square represents a magnified ROI. Scale bars, 42 μm. DAPI, 4′,6-diamidino-2-phenylindole; ROI, region of interest. Click here for file

Additional file 8 **Changes in autophagic factors in astrocytes.** Representative immunoblots show the immunoreactivity of **(A)** Beclin-1, **(B)** p62, **(C)** LC3-I, LC3-II, **(D)** mTOR, P_S2448_-mTOR, **(E)** p70S6K, P_T389_-p70S6K, and β-actin from cell lysates of primary astrocytes pretreated or not with 210 nM C16 and exposed or not to 20 μM Aβ42 in serum-free medium and treated with 200 pg/mL of IL-1β alone for 48 hours, treated or not with an autophagic flux inhibitor bafilomycin A1 (Baf) at 50 nM for 24 hours before cell lysis. Densities were quantified using GeneTools software. Data of each protein were reported to data of the corresponding β-actin. The results are expressed as arbitrary units (percentage of control set at 100%) and are mean ± SEM from six independent experiments in duplicate. ^*^P <0.05, ^**^P < 0.01, ^***^P <0.001 compared to control; ^§§^*P* <0.01, ^§§§^*P* < 0.001 compared to Aβ42; ^&^*P* <0.05, ^&&&^*P* <0.001 compared to C16; ^##^*P* <0.01, ^###^*P* <0.001 compared to IL-1β; ^$$$^*P* <0.001 compared to IL-1β with Aβ42 by one-way ANOVA with a Newman-Keuls multiple comparison test. Baf, bafilomycin A1. Click here for file
