# Supplementary material for: Involvement of interleukin-1β in the autophagic process of microglia: relevance to Alzheimer’s disease
Source: J Neuroinflammation. 2013 Dec 13;10:151. doi: 10.1186/1742-2094-10-151 (PMC3878742; doi:10.1186/1742-2094-10-151)
Supplement: Additional file 1 — Cytokine levels induced by inflammatory stress in autophagy inhibitory condition. Cytokine (IL1-β, TNF-α and IL-6) levels in culture medium (A, B, C, respectively) and in cell lysates (D, E, F, respectively) of tri-cultures in serum-free medium treated with an autophagic flux inhibitor, 50 nM bafilomycin A1 (Baf), with 100 ng/mL LPS or with 20 μM Aβ42 and pre-treated or not with 210 nM C16, were analyzed by the 3-plex Luminex xMAP assay containing a mixture of specific beads for each cytokine as described in the Methods section. Cytokine levels in culture medium and cell lysates are expressed in pg/mL and pg/mg protein, respectively. Results are mean ± SEM for six experiments in duplicate. ***P <0.001 compared to Baf alone; ###P <0.001 compared to LPS with Baf; †P <0.05, ††P <0.01, †††P <0.001 compared to Baf with C16 by one-way ANOVA with a Newman-Keuls multiple comparison test. Baf, bafilomycin A1; LPS, lipopolysaccharide. [file 1742-2094-10-151-S1.docx]

**B**

**C**

**A**

**D**

**F**

**E**

+

+

-

+

+

+

-

-

+

-

-

+

+

-

-

-

+

-

+

-

+

+

+

-

+

+

+

+

+

+

+

-

+

+

-

-

+

+

-

-

-

-

-

-

+

-

+

-

+

+

+

+

+

+

+

-

+

+

-

-

+

+

-

-

-

-

-

-

+

-

+

-

**LPS**

**Aβ42**

**C16**

**Baf**

**Additional file 1: Cytokine levels induced by inflammatory stress in autophagy inhibitory condition.** Cytokine (IL1-β, TNF-α and IL-6) levels in culture medium (A, B, C, respectively) and in cell lysates (D, E, F, respectively) of tri-cultures in serum-free medium treated with an autophagic flux inhibitor, 50 nM Bafilomycin A1, with 100 ng/mL LPS or with 20 µM Aβ42 and pre-treated or not with 210 nM C16, were analyzed by the 3-plex Luminex xMAP^®^ assay containing a mixture of specific beads for each cytokine as described in materials and methods. Cytokine levels in culture medium and in cell lysates were expressed in pg/mL and in pg/mg protein, respectively. Results are mean ± SEM for 6 experiments in duplicate. ^***^p < 0.001 compared to Baf alone; ^###^p < 0.001 compared to LPS with Baf; ^†^p < 0.05, ^††^p < 0.01, ^†††^p < 0.001 compared to Baf with C16 by one-way ANOVA with a Newman-Keuls multiple comparison test.
