# Supplementary material for: Involvement of interleukin-1β in the autophagic process of microglia: relevance to Alzheimer’s disease
Source: J Neuroinflammation. 2013 Dec 13;10:151. doi: 10.1186/1742-2094-10-151 (PMC3878742; doi:10.1186/1742-2094-10-151)
Supplement: Additional file 2 — Immunofluorescence of p62 and LC3 in neurons and astrocytes under inflammatory stress in primary tri-cultures. Co-labeling of autophagic factor (A) p62 (red) or (B) LC3 (red), MAP2 for neurons (green), GFAP for astrocytes (blue), and DAPI for nuclei (cyan) in tri-cultures seeded on coverslips and exposed to 20 μM Aβ42 or LPS 100 ng/mL in serum-free medium for 48 hours. All images were from a compilation of the entire z-series sections acquired by confocal microscopy (Olympus IX-81). A white square represents a magnified ROI. Scale bars, 42 μm. DAPI, 4′,6-diamidino-2-phenylindole; GFAP, glial fibrillary acidic protein; LPS, lipopolysaccharide; MAP2, microtubule-associated protein 2; ROI, region of interest. [file 1742-2094-10-151-S2.docx]

**B**

**A**

LPS


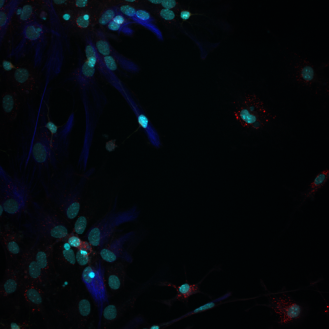


**LC3 Map-2 GFAP DAPI**


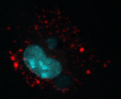


Control


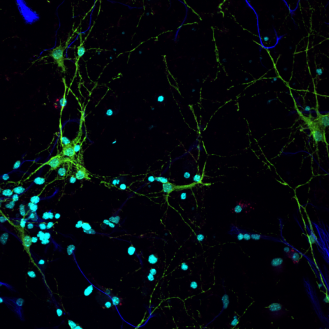


**LC3 Map-2 GFAP DAPI**


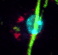

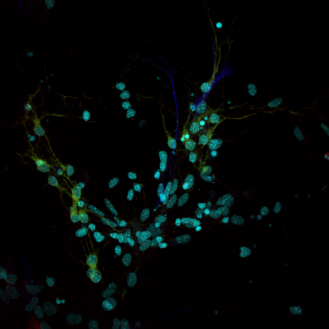


Aβ42

**LC3 Map-2 GFAP DAPI**


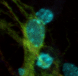


LPS


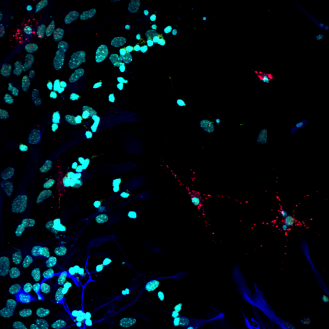


**p62 Map-2 GFAP DAPI**


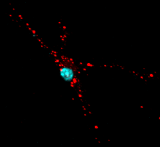


Aβ42


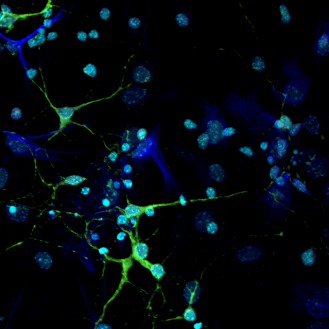


**p62 Map-2 GFAP DAPI**


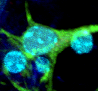


Control


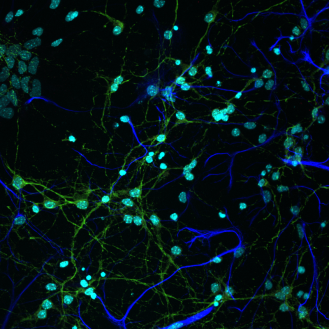


**p62 Map-2 GFAP DAPI**


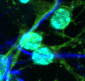


**Additional file 2: Immunofluorescence of p62 and LC3 in neurons and astrocytes under inflammatory stress in primary tri-cultures**. Co-labelling of autophagic factor p62 (A) and LC3 (B) in red, MAP-2 for neurons (green), GFAP for astrocytes (blue) and DAPI for nuclei (cyan) in tri-cultures seeded on coverslips and exposed to 20 μM Aβ42 or LPS 100 ng/mL in serum-free medium during 48hrs. All images were from a compilation of the entire Z-series sections acquired by confocal microscopy (Olympus IX-81). A white square represented a magnified ROI. Scale bars, 42 µm.
