# Supplementary material for: Involvement of interleukin-1β in the autophagic process of microglia: relevance to Alzheimer’s disease
Source: J Neuroinflammation. 2013 Dec 13;10:151. doi: 10.1186/1742-2094-10-151 (PMC3878742; doi:10.1186/1742-2094-10-151)
Supplement: Additional file 3 — Changes in autophagic factors under inflammatory stress. Representative immunoblots show the immunoreactivity of (A) p62, (B) LC3-I, LC3-II, and β-actin from cell lysates of primary tri-cultures exposed or not to 20 μM Aβ42 pretreated or not with 210 nM C16 in serum-free medium and treated with 200 pg/mL of TNF-α or IL-6 alone for 48 hours, treated or not with an autophagic flux inhibitor bafilomycin A1 (Baf) at 50 nM for 24 hours before cell lysis. Densities were quantified using GeneTools software. Data of each protein were reported to data of the corresponding β-actin. The results are expressed as arbitrary units (percentage of the control set at 100%) and are mean ± SEM from six independent experiments in duplicate. ***P <0.001 compared to control; ###P <0.001 compared to Baf by one-way ANOVA with a Newman-Keuls multiple comparison test. Baf, bafilomycin A1. [file 1742-2094-10-151-S3.docx]

**A**

--

---

---

---

---

--

---

---

+++

---

--

---

---

---

+++

--

---

---

+++

+++

--

---

+++

---

---

--

---

+++

---

+++

--

---

+++

+++

+++

--

+++

---

---

---

--

+++

---

---

+++

--

+++

---

+++

+++

++

--

--

--

--

++

--

--

++

--

++

--

--

--

++

++

--

--

++

++

++

--

--

++

+

++

--

++

++

+

++

--

--

--

+

--

+

+

+

-

--

+

+

-

+

--

+

+

+

+

--

+

+

-

-

--

---

---

+++

+++

--

---

+++

---

+++

++

--

--

+

++

**Aβ42**

**C16**

**TNF-α**

**IL-6**

**Baf**


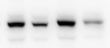

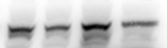

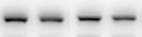

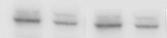

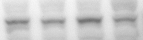

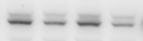


**p62**


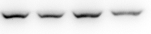

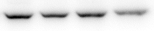

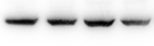

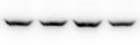

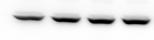

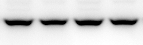


**β-actin**

+

-

-

-

+

+

-

-

+

+

+

-

-

+

+

+

-

+

+

+

-

+

+

-

+

-

+

+

+

-

-

+

+

-

-

-

+

+

+

+

-

+

+

-

+

+

+

-

-

+

-

-

+

+

+

-

-

-

+

+

+

-

-

-

+

+

+

-

-

+

-

-

**Aβ42**

**C16**

**TNF-α**

**IL-6**

**Baf**

**B**

-

+

-

-

-

-

-

-

-

+

+

+

-

-

-

-

+

-

-

-

-

+

-

+

-

+

-

+

+

-

+

-

+

-

-

-

-

-

-

-

-

-

-

-

-

-

-

-


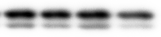

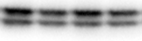

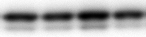

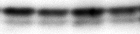

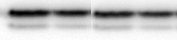

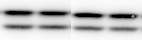


**LC3 I**

**LC3 II**


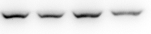

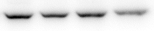

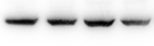

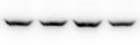

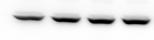

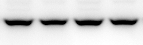


**β-actin**

**Additional file 3: Changes in autophagic factors under inflammatory stress.** Representative immunoblots showed the immunoreactivity of p62 (**A**), LC3 I, LC3 II (**B**) and β-actin from cell lysates of primary tri-cultures exposed or not to 20 μM Aβ42 pretreated or not with 210 nM C16 in serum-free medium and treated with 200 pg/mL of TNF-α or IL-6 alone during 48hrs, treated or not with an autophagic flux inhibitor Bafilomycin A1 at 50 nM 24hrs before cell lysis. Densities were quantified by using Gene Tools software (Syngene, Ozyme France). Data of each protein were reported to data of the corresponding β-actin. The results are expressed as arbitrary units (% of control set at 100%) and are mean ± SEM from 6 independent experiments in duplicate. ^***^p < 0.001 compared to control; ^###^p < 0.001 compared to Baf by one-way ANOVA with a Newman-Keuls multiple comparison test.
