# Supplementary material for: Involvement of interleukin-1β in the autophagic process of microglia: relevance to Alzheimer’s disease
Source: J Neuroinflammation. 2013 Dec 13;10:151. doi: 10.1186/1742-2094-10-151 (PMC3878742; doi:10.1186/1742-2094-10-151)
Supplement: Additional file 4 — Activation of mTOR signaling pathway by exogenous cytokines in tri-cultures. Representative immunoblots show the immunoreactivity of (A) mTOR, PS2448-mTOR, (B) p70S6K, PT389-p70S6K, and β-actin from cell lysates of primary tri-cultures exposed to 20 μM Aβ42, pretreated or not with 210 nM C16 in serum-free medium and treated with a cytokine cocktail of 200 pg/mL of IL-1β, TNF-α, and IL-6 or with 200 pg/mL of IL-1β, TNF-α, or IL-6 alone for 48 hours. Densities were quantified using GeneTools software. Data of each protein were reported to data of the corresponding β-actin. The results are expressed as arbitrary units (percentage of the control set at 100%) and are mean ± SEM from six independent experiments in duplicate. ***P <0.001 compared to control; &&P <0.01, &&&P <0.001 compared to C16; ‡‡P <0.01, ‡‡‡P <0.001 compared to inflammatory cocktail; $P <0.05 compared to TNF-α; §§§P <0.001 compared to TNF-α with C16; †P <0.05 compared to IL-6; δP <0.05 compared to IL-6 with C16 by one-way ANOVA with a Newman-Keuls multiple comparison test. mTOR, mammalian target of rapamycin. [file 1742-2094-10-151-S4.docx]

+

-

-

-

-

-

-

+

-

-

-

+

-

-

+

-

-

-

-

+

-

+

-

-

-

+

+

-

+

-

-

+

-

-

-

+

-

+

+

-

-

+

-

+

-

-

-

-

+

-

-

+

-

-

-

+

+

+

-

-

-

-

-

-

+

-

-

-

+

+

-

-

-

-

-

+

-

+

-

-

-

-

+

+

+

-

-

-

+

+

-

-

-

-

-

-

-

-

-

-

+

-

-

-

-

+

-

-

-

-

-

-

-

-

-

+

-

-

-

-

-

-

-

+

-

-

-

-

-

+

-

+

-

-

-

-

-

-

-

-

**Aβ42**

**C16**

**Cocktail**

**IL-1β**

**TNF-α**

**IL-6**

**Baf**

**A**


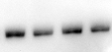

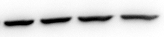

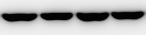

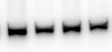

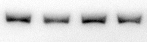

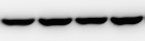

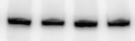

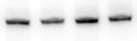

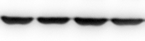

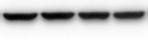

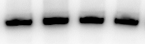

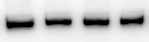

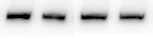

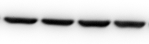

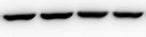

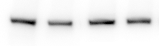

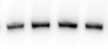

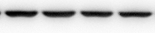

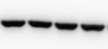

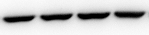


**P_S2448_-mTOR**

**mTOR**

**β-actin**

**β-actin**

-

+

-

+

+

-

-

-

+

-

-

+

-

-

+

-

-

-

+

-

-

**B**

**Aβ42**

**C16**

**Cocktail**

**IL-1β**

**TNF-α**

**IL-6**

**Baf**

-

-

+

+

+

-

-

-

-

+

-

+

-

-

-

-

+

-

-

+

-

-

-

-

-

-

-

+

-

-

-

-

+

-

+

-

+

-

-

-

-

-

+

-

-

-

-

+

-

+

-

-

-

-

-

-

+

-

-

-

+

+

-

-

-

-

+

+

+

-

-

-

-

+

+

-

-

-

-

-

+

-

+

-

-

-

-

+

-

-

-

-

-

-

+

+

-

-

-

-

-

-

+

-

-

-

-

-

-

-

+

-

-

-

-

-

-

-

-


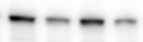

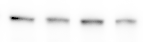

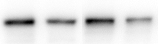

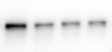

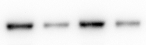


**β-actin**

**P_T389_-p70S6K**

**p70S6K**

**β-actin**


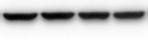

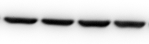

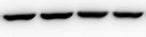

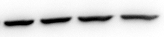

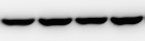


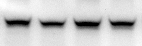

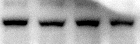

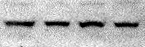

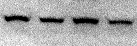

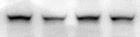


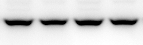

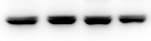

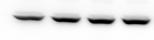

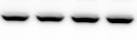

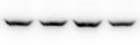

**Additional file 4: Activation of mTOR signaling pathway by exogenous cytokines in tri-cultures.** Representative immunoblots showed the immunoreactivity of mTOR, P_S2448_-mTOR (A) and p70S6K, P_T389_-p70S6K (B), and β-actin from cell lysates of primary tri-cultures exposed to 20 μM Aβ42, pretreated or not with 210 nM C16 in serum-free medium and treated with a cytokine cocktail of 200 pg/mL of IL-1β, TNF-α and IL-6 or with 200 pg/mL of IL-1β, TNF-α or IL-6 alone during 48hrs. Densities were quantified by using Gene Tools software (Syngene, Ozyme France). Data of each protein were reported to data of the corresponding β-actin. The results are expressed as arbitrary units (% of control set at 100%) and are mean ± SEM from 6 independent experiments in duplicate. ^***^p < 0.001 compared to control; ^&&^p<0.01, ^&&&^p<0.001 compared to C16; ^‡‡^p<0.01, ^‡‡‡^p<0.001 compared to inflammatory cocktail; ^$^p<0.05 compared to TNF-α; ^§§§^p<0.001 compared to TNF-α with C16; ^†^p<0.05 compared to IL-6; ^δ^p<0.05 compared to IL-6 with C16 by one-way ANOVA with a Newman-Keuls multiple comparison test.
