# Supplementary material for: Involvement of interleukin-1β in the autophagic process of microglia: relevance to Alzheimer’s disease
Source: J Neuroinflammation. 2013 Dec 13;10:151. doi: 10.1186/1742-2094-10-151 (PMC3878742; doi:10.1186/1742-2094-10-151)
Supplement: Additional file 5 — Cytokine levels induced by exogenous TNF-α and IL-6. Cytokine (TNF-α and IL-6) levels in culture medium (A and B, respectively) of tri-cultures pre-treated or not with 210 nM C16, exposed or not to 20 μM Aβ42, and treated with 200 pg/mL of TNF-α or IL-6 alone in serum-free medium were analyzed by the 3-plex Luminex xMAP assay containing a mixture of specific beads for each cytokine as described in the Methods section. Cytokine levels are expressed in pg/mL. Results are mean ± SEM for six experiments in duplicate. **P <0.01, ***P <0.001 compared to respective control by one-way ANOVA with a Newman-Keuls multiple comparison test. [file 1742-2094-10-151-S5.docx]

**B**

**A**

**Aβ42**

**C16**

**TNF-α**

**IL-6**

-

-

-

-

-

-

+

-

-

-

-

+

-

-

+

+

-

+

-

-

-

+

-

+

-

+

+

+

-

-

+

+

+

-

+

+

-

+

-

+

+

-

-

+

+

-

-

-

---

-

---

---

-

---

+++

---

---

-

---

+++

-

---

+++

+++

-

+++

---

---

-

+++

---

+++

-

+++

+++

+++

---

---

+++

+++

+

---

+++

+++

---

+++

---

+

+++

---

---

+++

+++

---

---

---

**Aβ42**

**C16**

**TNF-α**

**IL-6**

**Additional file 5: Cytokine levels induced by exogenous TNF-α and IL-6.** Cytokine (TNF-α and IL-6) levels in culture medium (A and B, respectively) of tri-cultures pre-treated or not with 210 nM C16, exposed or not to 20 μM Aβ42, and treated with 200 pg/mL of TNF-α or IL-6 alone in serum-free medium were analyzed by the 3-plex Luminex xMAP^®^ assay containing a mixture of specific beads for each cytokine as described in materials and methods. Cytokine levels were expressed in pg/mL. Results are mean ± SEM for 6 experiments in duplicate. ^**^p < 0.01, ^***^p < 0.001 compared to respective control by one-way ANOVA with a Newman-Keuls multiple comparison test.
