# Supplementary material for: Involvement of interleukin-1β in the autophagic process of microglia: relevance to Alzheimer’s disease
Source: J Neuroinflammation. 2013 Dec 13;10:151. doi: 10.1186/1742-2094-10-151 (PMC3878742; doi:10.1186/1742-2094-10-151)
Supplement: Additional file 6 — Cytokine levels induced by inflammatory stress in astrocytes. Cytokine (IL-1β and IL-6) levels in cell lysates (A and B, respectively) and in culture medium (C and D, respectively) of primary astrocytes pre-treated or not with 210 nM C16, exposed or not to 20 μM Aβ42, and treated with 200 pg/mL of IL-1β alone in serum-free medium were analyzed by the 3-plex Luminex xMAP assay containing a mixture of specific beads for each cytokine as described in the Methods section. Cytokine levels in cell lysates and culture medium are expressed in pg/mg protein and pg/mL, respectively. Results are mean ± SEM for six experiments in duplicate. ***P <0.001 compared to respective control; ††P <0.01, †††P <0.001, compared to IL-1β; ϵϵP <0.01, ϵϵϵP <0.001 compared to Aβ42 with IL-1β by one-way ANOVA with a Newman-Keuls multiple comparison test. [file 1742-2094-10-151-S6.docx]

**B**

**A**

**D**

**C**

**Aβ42**

**C16**

**IL-1β**

**Aβ42**

**C16**

**IL-1β**

+

+

+

-

+

+

+

-

+

+

-

-

-

+

+

-

-

+

-

+

-

-

-

-

+

+

+

-

+

+

+

-

+

+

-

-

-

+

+

-

-

+

-

+

-

-

-

-

**Additional file 6: Cytokine levels induced by inflammatory stress in astrocytes**. Cytokine (IL-1β and IL-6) levels in cell lysates (A, B respectively) and in culture medium (C, D respectively) of primary astrocytes pre-treated or not with 210 nM C16, exposed or not to 20 μM Aβ42, and treated with 200 pg/mL of IL-1β alone in serum-free medium were analyzed by the 3-plex Luminex xMAP^®^ assay containing a mixture of specific beads for each cytokine as described in materials and methods. Cytokine levels in cell lysates and in culture medium were expressed in pg/mg protein and in pg/mL, respectively. Results are mean ± SEM for 6 experiments in duplicate. ^***^p < 0.001 compared to respective control, ^††^p < 0.01, ^†††^p < 0.001, compared to IL-1β, ^εε^p < 0.01, ^εεε^p < 0.001 compared to Aβ42 with IL-1β by one-way ANOVA with a Newman-Keuls multiple comparison test.
