# Supplementary material for: Involvement of interleukin-1β in the autophagic process of microglia: relevance to Alzheimer’s disease
Source: J Neuroinflammation. 2013 Dec 13;10:151. doi: 10.1186/1742-2094-10-151 (PMC3878742; doi:10.1186/1742-2094-10-151)
Supplement: Additional file 7 — Immunostaining of p62 or LC3 and Lyso-ID in purified microglia. Co-labeling of the autophagic receptor (A) p62 (green) or (B) LC3 (green), Lyso-ID (red), and DAPI for nuclei (cyan) in microglia seeded on coverslips and exposed to 20 μM Aβ42 or 200 pg/mL IL-1β in serum-free medium for 48 hours. All images were from a compilation of the entire z-series sections acquired by confocal microscopy (Olympus IX-81). A white square represents a magnified ROI. Scale bars, 42 μm. DAPI, 4′,6-diamidino-2-phenylindole; ROI, region of interest. [file 1742-2094-10-151-S7.docx]

A


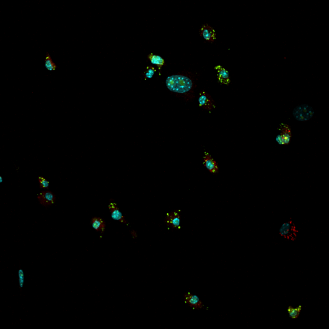


**p62 LD DAPI**


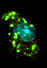

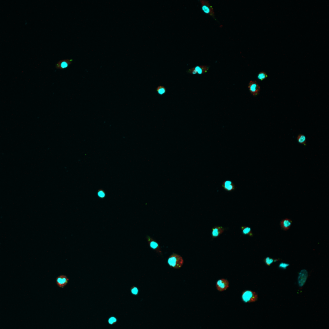

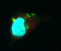


**p62 LD DAPI**


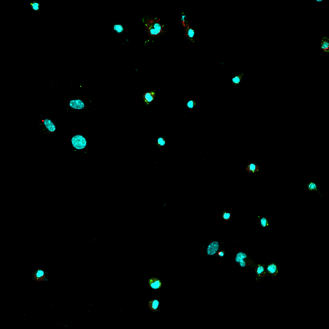

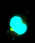


**p62 LD DAPI**


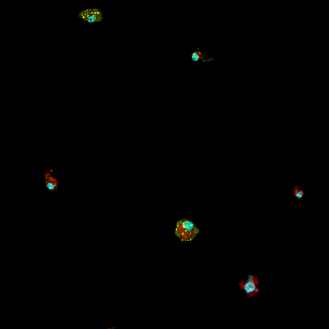

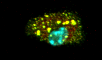


**p62 LD DAPI**

IL-1β

IL-1β + Aβ42

Aβ42

Control

B


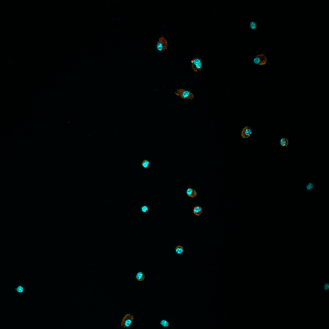

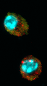


**LC3 LD DAPI**


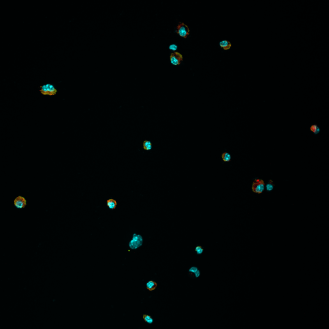


**LC3 LD DAPI**


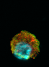

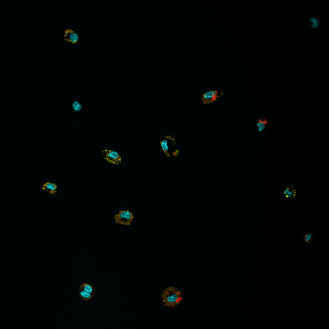

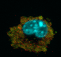


**LC3 LD DAPI**


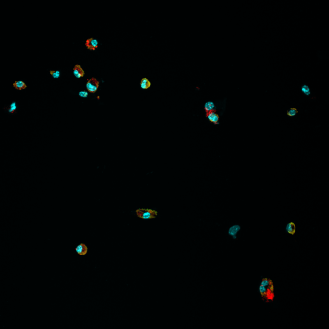

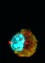


**LC3 LD DAPI**

**Additional file 7: Immunostaining of p62 or LC3 and Lyso-ID^®^ in purified microglia.** Co-labelling of the autophagic receptor p62 (green, panel A) or LC3 (green, panel B), Lyso-ID^®^ (red) and DAPI for nuclei (cyan) in microglia seeded on coverslips and exposed to 20 μM Aβ42 or 200 pg/mL IL-1β in serum-free medium during 48hrs. All images were from a compilation of the entire Z-series sections acquired by confocal microscopy (Olympus IX-81). A white square represented a magnified ROI. Scale bars, 42 µm.
